# KSHV vIL-6 promotes SIRT3-induced deacetylation of SERBP1 to inhibit ferroptosis and enhance cellular transformation by inducing lipoyltransferase 2 mRNA degradation

**DOI:** 10.1371/journal.ppat.1012082

**Published:** 2024-03-12

**Authors:** Jing Zhou, Tianjiao Wang, Haoran Zhang, Jianhong Liu, Pengjun Wei, Ruoqi Xu, Qin Yan, Guochun Chen, Wan Li, Shou-Jiang Gao, Chun Lu

**Affiliations:** 1 Department of Microbiology, Nanjing Medical University, Nanjing, People’s Republic of China; 2 Department of Pathology, Changzhou Third People’s Hospital, Changzhou, People’s Republic of China; 3 Changzhou Medical Center, Nanjing Medical University, Nanjing, People’s Republic of China; 4 Key Laboratory of Pathogen Biology of Jiangsu Province, Nanjing Medical University, Nanjing, People’s Republic of China; 5 Department of Infectious Diseases, Changzhou Third People’s Hospital, Changzhou, People’s Republic of China; 6 Tumor Virology Program, UPMC Hillman Cancer Center, and Department of Microbiology and Molecular Genetics, University of Pittsburgh, Pittsburgh, Pennsylvania, United States of America; Harvard University, UNITED STATES

## Abstract

Ferroptosis, a defensive strategy commonly employed by the host cells to restrict pathogenic infections, has been implicated in the development and therapeutic responses of various types of cancer. However, the role of ferroptosis in oncogenic Kaposi’s sarcoma-associated herpesvirus (KSHV)-induced cancers remains elusive. While a growing number of non-histone proteins have been identified as acetylation targets, the functions of these modifications have yet to be revealed. Here, we show KSHV reprogramming of host acetylation proteomics following cellular transformation of rat primary mesenchymal precursor. Among them, SERPINE1 mRNA binding protein 1 (SERBP1) deacetylation is increased and required for KSHV-induced cellular transformation. Mechanistically, KSHV-encoded viral interleukin-6 (vIL-6) promotes SIRT3 deacetylation of SERBP1, preventing its binding to and protection of lipoyltransferase 2 (Lipt2) mRNA from mRNA degradation resulting in ferroptosis. Consequently, a SIRT3-specific inhibitor, 3-TYP, suppresses KSHV-induced cellular transformation by inducing ferroptosis. Our findings unveil novel roles of vIL-6 and SERBP1 deacetylation in regulating ferroptosis and KSHV-induced cellular transformation, and establish the vIL-6-SIRT3-SERBP1-ferroptosis pathways as a potential new therapeutic target for KSHV-associated cancers.

## Author summary

Ferroptosis has been implicated in viral infections and the development of cancers, however, its role in oncogenic Kaposi’s sarcoma-associated herpesvirus (KSHV)-induced cancers is still unknown. Although abnormal protein lysine acetylation modification has been strongly implicated in tumorigenesis, the exact role of this modification in KSHV-induced cell transformation remains elusive. Our study uncovers the reduction of acetylation of SERPINE1 mRNA binding protein 1 (SERBP1), which is required for KSHV-induced cellular transformation. KSHV-encoded viral interleukin-6 (vIL-6) promotes SIRT3 deacetylation of SERBP1, preventing its binding to and protection of lipoyltransferase 2 (Lipt2) mRNA from mRNA degradation resulting in ferroptosis. A SIRT3-specific inhibitor, 3-TYP, induces ferroptosis and inhibits survival of KSHV-transformed cells. These findings define a novel mechanism contributing to KSHV-induced cellular transformation and provide a rationale for therapeutically targeting lysine acetylation and the associated enzyme for infectious diseases.

## Introduction

Kaposi’s sarcoma-associated herpesvirus (KSHV) was identified by Chang et al. in 1994 from a Kaposi’s sarcoma sample from a patient with acquired immunodeficiency syndrome-related Kaposi’s sarcoma (AIDS-KS) [1]. KSHV, belonging to the γ-herpesvirus, possesses a double stranded DNA genome spanning a total length of around 140 kb and encompasses approximately 90 open reading frames (ORFs) [2]. In addition to its association with KS, KSHV infection is implicated in the development of three other malignancies, namely primary effusion lymphoma (PEL), a subset of multicenter Castleman’s disease (MCD) and KS inflammatory cytokine syndrome (KICS) [3,4]. Similar to other herpesviruses, KSHV undergoes two distinct phases of life cycle: lytic replication and latent infection, both of which play pivotal roles in KSHV-induced malignancies [5,6]. The onset of the lytic phase in KSHV infection is characterized by the initial expression of the replication and transcription activator (RTA/ORF50). Subsequently, RTA facilitates the sequential expression of other downstream lytic genes, such as viral interleukin 6 (vIL-6) [7]. During KSHV latency, only a limited subset of genes is expressed, including latency associated nuclear antigen (LANA) encoded by ORF73, viral cyclin (vCyclin) encoded by ORF72, viral FADD-like interleukin (IL)-1-β-converting enzyme (FLICE/caspase-8) inhibitory protein (vFLIP) encoded by ORF71/K13, Kaposin protein encoded by K12, as well as 25 mature microRNAs [8–10]. These key genes play an indispensable role in the initiation and progression of KSHV-induced tumors.

Diverse pathways of regulated cell death (RCD) exert distinct impacts on tumor progression [11]. Among the extensively investigated forms of RCD are apoptosis, pyroptosis, necroptosis and ferroptosis [12]. Ferroptosis is an iron dependent regulatory cell death form caused by excessive lipid peroxidation, which has been proven as a contributing factor in the initiation and advancement of various cancer types, potentially influencing cancer initiation and progression [13]. Ferroptosis can be triggered via two major pathways: the extrinsic or transporter-dependent pathway, and the intrinsic or enzyme-regulated pathway [14]. Augmented iron accumulation, free radical generation, fatty acid surplus and lipid peroxidation facilitated by dedicated enzymes are critical for the induction of ferroptosis. Multiple oxidative and antioxidant systems, working in concert with the autophagy and membrane repair machinery, orchestrate the process of lipid peroxidation during ferroptosis [15]. However, it is unclear whether ferroptosis is involved in KSHV-induced tumorigenesis.

Protein post-translational modifications (PTMs) refer to the covalent addition of functional groups to proteins after protein translation. PTMs represent a complex process that expands the repertoire of protein types, enhances their functionalities, and actively participates in nearly all cellular processes, exerting crucial regulatory roles [16]. In the early 1960s, histone acetylation emerged as the first identified regulator of gene transcription [17,18]. Subsequently, numerous non-histone proteins were identified as targets for acetylation [19,20]. Acetyl-CoA, a central metabolite and substrate for anabolic metabolism, serves as the donor of protein lysine acetylation (Kac), with its production occurring in the mitochondria, cytoplasm, or nucleus [21]. All lysine acetyltransferases (KATs) engaged in acetylation reactions require acetyl-CoA as donor. Consequently, protein lysine acetylation represents an important mechanism governing overall energy metabolisms through its influence on metabolic enzymes and transcription factors [21]. Indeed, non-histone protein acetylation is involved in various cellular processes relevant to physiology and disease, such as gene transcription, DNA damage repair, cell division, signal transduction, protein folding, autophagy, and metabolism. Acetylation exerts its effects on protein functionalities through diverse mechanisms, including the regulation of protein stability, enzymatic activity, subcellular localization and so on [22]. Our previous studies have shown that KSHV vIL-6 enhances the acetylation of STAT3 to induce angiogenesis and cellular transformation [23]. We also found that KSHV vFLIP promotes cell invasion and angiogenesis by regulating p65 acetylation [24]. These findings suggest that non-histone proteins acetylation plays an important role in KSHV-induced tumorigenesis.

In this study, we sought to identify the differential non-histone acetylated proteins in KSHV-transformed cells and investigate their effects on KSHV-induced cellular transformation. We uncovered a reprogrammed acetylation proteomics in KSHV-transformed rat primary mesenchymal precursor cells (KMM) and observed that alteration in the acetylation of SERPINE1 mRNA binding protein 1 (SERBP1) impacted ferroptosis in KMM cells. Subsequently, we revealed that KSHV vIL-6 recruited SIRT3 to bind to SERBP1, leading to its deacetylation and subsequent inhibition of ferroptosis and enhancement of KSHV-induced cellular transformation. Deacetylated SERBP1 prevented its binding to lipoyltransferase 2 (Lipt2) mRNA, which resulted in Lipt2 mRNA degradation and inhibition of ferroptosis. A SIRT3-specific inhibitor, 3-TYP, inhibited KSHV-induced cellular transformation by inducing ferroptosis. Our findings reveal a novel mechanism by which SERBP1 deacetylation contributes to KSHV-induced cellular transformation by inhibiting ferroptosis, and thus providing insights into the molecular basis of KSHV-induced tumorigenesis.

## Results

### SERBP1 deacetylation is required for efficient KSHV-induced cellular transformation

**Fig 1:**
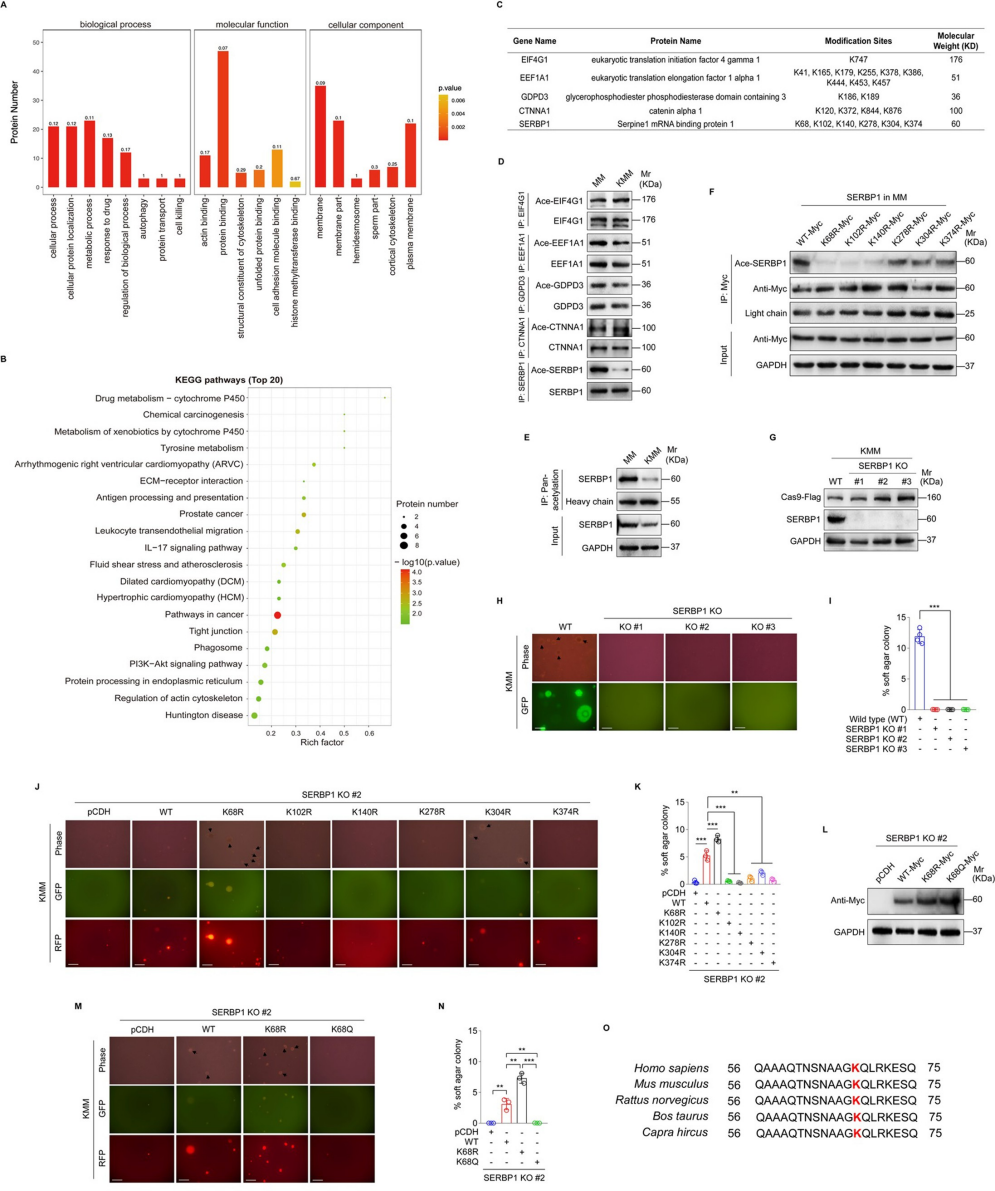
SERBP1 deacetylation contributes to KSHV-induced cellular transformation. **(A).** GO enrichment analyses of the acetylated proteins in MM and KMM cells. **(B).** KEGG pathway of the acetylated proteins in MM and KMM cells. P-value for enrichment present the only pathways that have statistical significance. **(C).** Differentially acetylated proteins obtained in 3 paired samples of MM and KMM cells. **(D).** The acetylation of differentially acetylated proteins in MM and KMM cells was examined by immunoprecipitating with corresponding antibody. **(E).** The acetylation level of SERBP1 in MM and KMM cells was examined by immunoprecipitating and Western blotting with anti-pan-acetylation lysine antibody and anti-SERBP1 antibody, respectively. **(F).** MM cells were transduced by six mutants of putative lysine residues in SERBP1. Acetylation levels of SERBP1 were determined by immunoprecipitating and Western blotting with anti-pan-acetylation lysine antibody and anti-SERBP1 antibody, respectively. **(G).** Western blotting analysis of SERBP1 in three SERBP1 knockout monoclonal KMM cell lines (**SERBP1 KO #1, KO #2, and KO #3**) generated by the CRISPR-Cas9 method. **(H).** Soft agar assay of cells treated as in (**G**). The representative images were captured at 2 weeks post seeding. Magnification, ×100. Scar bars, 40 μm. The representative images were taken from three randomly selected fields of each sample. **(I).** Quantification of the results in (**H**). Colonies with a size equal to or larger than 20 μm (arrows shown in **H**) were counted to calculate the percentage of soft agar colonies. **(J).** Soft agar assay was performed in SERBP1 knockout KMM cells (**SERBP1 KO #2**) transduced with six mutants of putative lysine residues in SERBP1. The representative images were captured at 2 weeks post seeding. Magnification, ×100. Scar bars, 40 μm. The representative images were taken from three randomly selected fields of each sample. **(K).** Quantification of the results in (**J**). Colonies with a size equal to or larger than 20 μm (arrows shown in **J**) were counted to calculate the percentage of soft agar colonies. **(L).** SERBP1 KO KMM cells (**SERBP1 KO #2**) were transduced by the wild type SERBP1 (**WT**), the deacetylation mutant form of SERBP1 (**K68R**) or the acetylation mutant form of SERBP1 (**K68Q**). The expression level of SERBP1 was examined by Western blotting with anti-Myc antibody. **(M).** Soft agar assay of cells treated as in (**L**). The representative images were captured at 2 weeks post seeding. Magnification, ×100. Scar bars, 40 μm. The representative images were taken from three randomly selected fields of each sample. **(N).** Quantification of the results in (**M**). Colonies with a size equal to or larger than 20 μm (arrows shown in **M**) were counted to calculate the percentage of soft agar colonies. **(O).** The amino acid sequences of SERBP1 from different species (Homo sapiens, Mus musculus, Rattus norvegicus, Bos taurus, Capra hircus) were compared. The acetylation site of SERBP1 was indicated in red. Data were shown as mean ± SD unless indicated else. **P < 0.01 and ***P < 0.001, Student’s *t*-test.

The KMM system is a robust model for studying KSHV-induced cellular transformation [25]. Here, we used this model to explore the acetylated proteins in KMM cells in comparison with the matched primary uninfected cells (MM). We performed a tandem mass tag (TMT) labeling proteomic screening and obtained a set of proteins with differential acetylation between MM and KMM cells. Then, we conducted enrichment analyses based on gene ontology (GO) annotation, encompassing biological processes, molecular functions, cellular components, and Kyoto Encyclopedia of Genes and Genomes (KEGG). Enrichment score with p-value was used to define specific pathway involvement. We found that the differential acetylated proteins were enriched in various signaling pathways. Among them, pathways associated with tumorigenesis enriched the highest number of proteins and the most significant statistical differences (Fig 1A and 1B). Subsequently, we chose five proteins exhibiting the most significant differences in acetylation modification for further verification (Fig 1C). Immunoprecipitation assay (IP) was performed to analyze lysine acetylation (Kac) using an acetylated-lysine antibody. After enriching SERBP1, we observed a decrease in SERBP1 acetylation in KMM cells compared to MM cells (Fig 1D). To further investigate the acetylation status of SERBP1, we used an anti-pan-acetylation lysine antibody to enrich acetylated proteins and then quantified SERBP1 levels. Consistent with the above observation, SERBP1 acetylation is reduced in KMM cells compared to MM cells (Fig 1E).

Proteomic analysis identified that SERBP1 has six potential acetylation sites: K68, K102, K140, K278, K304, and K374 (Figs 1C and S1). We mutated each site by replacing a lysine (K) with an arginine (R) to mimic a constitutively deacetylated state, and validated the effect of these mutations on SERBP1 acetylation level. We showed that all these six mutations reduced the level of SERBP1 acetylation, particularly, K68R, K102R and K140R, which caused the most obvious changes (Fig 1F). Then, we utilized the CRISPR-Cas9 technology to generate three SERBP1 knockout (KO) clonal cell lines (Fig 1G). The colony formation assay in soft agar demonstrated a complete elimination of colony formation of KMM cells upon SERBP1 knockout (Fig 1H and 1I). This deficiency could be rescued by overexpression of wild type SERBP1 (WT), as well as K68R mutant of SERBP1 (K68R), but not other five KR mutants of SERBP1 (Fig 1J and 1K), indicating that K68 was the major acetylation site of SERBP1 mediating colony formation in soft agar. We further generated a SERBP1 lysine (K)-to-glutamine (Q) mutation at K68 to mimic the activated acetylation in SERBP1 (Fig 1L). Intriguingly, K68Q mutant significantly weakened the colony formation ability of KMM cells (Fig 1M and 1N). Sequence alignment showed the high conservation of K68 in SERBP1 from different species (Fig 1O). These findings collectively provide compelling evidence that the deacetylation of SERBP1 at K68 contributes to KSHV-induced cellular transformation.

### SERBP1 deacetylation inhibits ferroptosis

**Fig 2:**
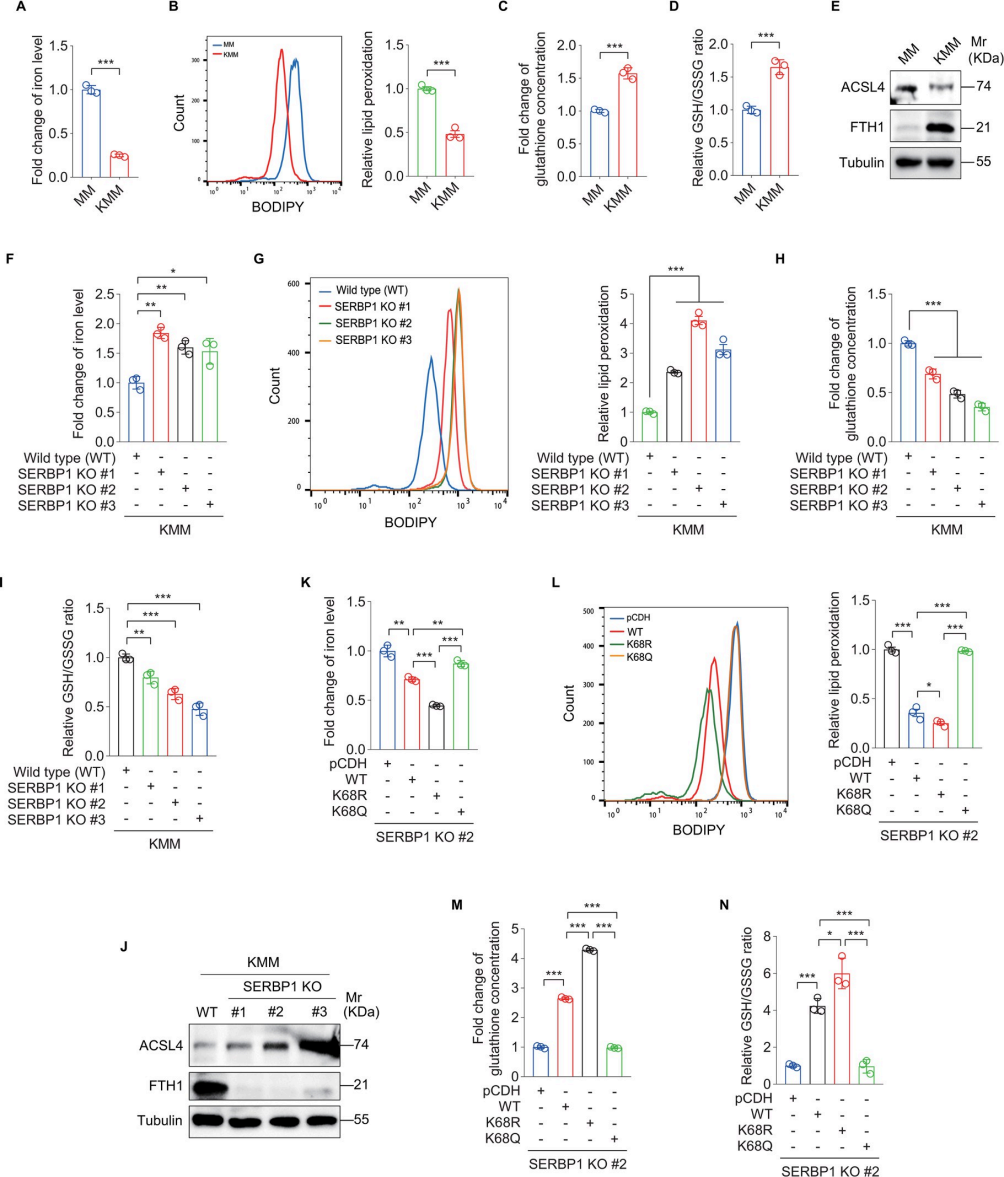
SERBP1 deacetylation mediates KSHV-induced cellular transformation by inhibiting ferroptosis. **(A).** Levels of iron in MM and KMM cells. **(B).** Flow cytometry analysis of lipid peroxidation level in MM and KMM cells (**left**). The lipid peroxidation level of indicated cells was shown in bar graph (**right**). Data represented the mean ± SEM of three independent experiments. **(C).** Levels of total glutathione in MM and KMM cells. **(D).** Levels of GSH/GSSG ratio in MM and KMM cells. **(E).** Western blotting analysis of ACSL4 and FTH1 in MM and KMM cells. **(F).** Levels of iron in three SERBP1 knockout monoclonal KMM cell lines (**SERBP1 KO #1, KO #2,** and **KO #3**). **(G).** Flow cytometry analysis of lipid peroxidation level in three SERBP1 knockout monoclonal KMM cell lines (**SERBP1 KO #1, KO #2, and KO #3**) (**left**). The lipid peroxidation level of indicated cells was shown in bar graph (**right**). Data represented the mean ± SEM of three independent experiments. **(H).** Levels of total glutathione in three SERBP1 knockout monoclonal KMM cell lines (**SERBP1 KO #1, KO #2,** and **KO #3**). **(I).** Levels of GSH/GSSG ratio in three SERBP1 knockout monoclonal KMM cell lines (**SERBP1 KO #1**, **KO #2**, and **KO #3**). **(J).** Western blotting analysis of ACSL4 and FTH1 in three SERBP1 knockout monoclonal KMM cell lines (**SERBP1 KO #1**, **KO #2**, and **KO #3**). **(K).** Levels of iron in SERBP1 knockout KMM cells (**SERBP1 KO #2**) transduced with lentiviral wild type SERBP1 (**WT**), K68R mutant SERBP1 (**K68R**), K68Q mutant SERBP1 (**K68Q**) or its control lentivirus pCDH (**pCDH**). **(L).** Flow cytometry analysis of lipid peroxidation level in cells treated as in (**K**) (**left**). The lipid peroxidation level of indicated cells was shown in bar graph (**right**). Data represented the mean ± SEM of three independent experiments. **(M).** Levels of total glutathione in cells treated as in (**K**). **(N).** Levels of GSH/GSSG ratio in cells treated as in (**K**). Data were shown as mean ± SD unless indicated else. *P < 0.05, **P < 0.01, and ***P < 0.001, Student’s *t*-test.

Ferroptosis is a newly discovered form of RCD characterized by the lethal accumulation of iron and reactive oxygen species (ROS)-dependent lipid hydroperoxides [15]. To determine the role of ferroptosis in KSHV-induced cellular transformation, we detected ferroptosis-related indicators in MM and KMM cells, including iron concentration, lipid peroxidation level, total glutathione concentration, and GSH/GSSG ratio (reduced to oxidized glutathione ratio). We observed a significant decrease in both iron concentration and lipid peroxidation level in KMM cells when compared to MM cells (Fig 2A and 2B). Meanwhile, we found an increase in glutathione concentration and GSH/GSSG ratio in KMM cells (Fig 2C and 2D). Further, we examined the expression of ferroptosis-related proteins. Downregulation of acyl-CoA synthetase long-chain family member 4 (ACSL4) and upregulation of ferritin heavy chain 1 (FTH1) were observed in KMM cells (Fig 2E). To confirm that ferroptosis contributes to KSHV-induced cellular transformation, we treated the KMM cells with Fer-1, a ferroptosis inhibitor. We found that Fer-1 treatment enhanced the efficiency of colony formation of KMM cells in soft agar (S2 Fig).

Next, we investigated whether SERBP1 deacetylation is involved in KSHV inhibition of ferroptosis. We observed an increase in iron concentration and lipid peroxidation level and a decrease in glutathione concentration and GSH/GSSG ratio in SERBP1 KO KMM cells (Fig 2F–2J). We further performed rescue experiments by reintroducing SERBP1 wild type (WT), K68R, or K68Q mutant into SERBP1 KO cells. We observed that deacetylated SERBP1 at K68 (K68R) dramatically inhibited ferroptosis. In contrast, acetylated SERBP1 at K68 (K68Q) had increased level of ferroptosis (Fig 2K–2N).

### SIRT3 induces SERBP1 deacetylation contributing to KSHV-induced cellular transformation by suppressing ferroptosis

**Fig 3:**
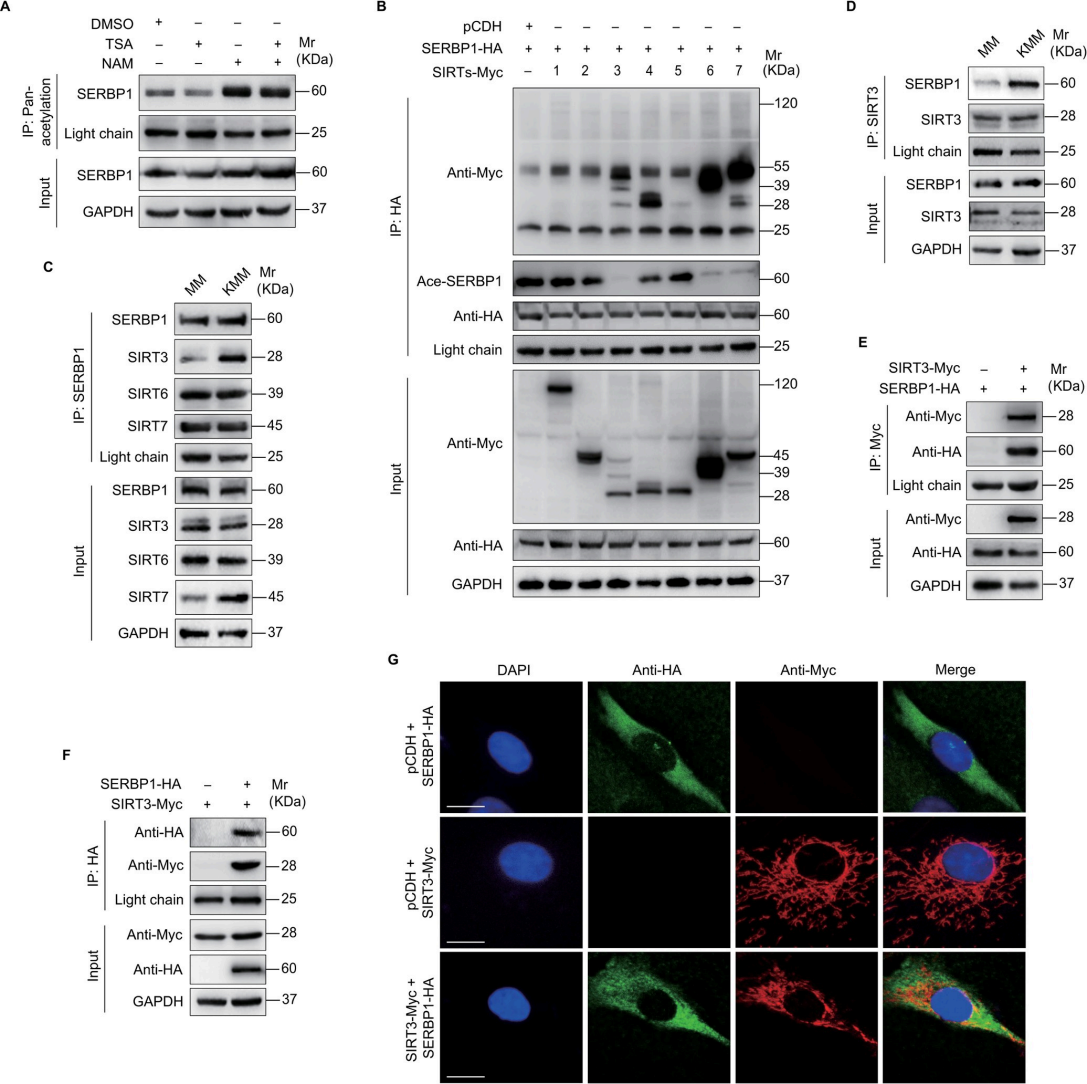
SIRT3 is a deacetylase, which deacetylates SERBP1. **(A).** KMM cells were treated with TSA or NAM for 8 h and then harvested. Acetylation levels of SERBP1 were examined by immunoprecipitating and Western blotting using an anti-pan-acetylation lysine antibody and anti-SERBP1 antibody, respectively. **(B).** KMM cells infected with lentiviral SERBP1-HA (**SERBP1-HA**) were transduced with lentiviral SIRT1-7 or its control pCDH (**pCDH**). The interaction between SERBP1 and SIRT1-7 proteins was examined by immunoprecipitating with anti-HA antibody. The acetylation of SERBP1 was examined by immunoblotting with anti-acetylated-lysine antibody. **(C).** The interaction between SERBP1 and SIRT3, 6, and 7 proteins was examined by immunoprecipitating with anti-SERBP1 antibody and immunoblotting with anti-SIRT3, 6, and 7 antibodies, respectively, in MM and KMM cells. **(D).** The interaction between SIRT3 and SERBP1 proteins was examined by immunoprecipitating with anti-SIRT3 antibody in MM and KMM cells. **(E).** KMM cells infected with lentiviral SERBP1-HA (**SERBP1-HA**) were transduced with lentiviral SIRT3-Myc (**SIRT3-Myc**) or its control pCDH (**pCDH**). The interaction between SIRT3 and SERBP1 proteins was examined by immunoprecipitating with anti-Myc antibody. **(F).** KMM cells infected with lentiviral SIRT3-Myc (**SIRT3-Myc**) were transduced with lentiviral SERBP1-HA (**SERBP1-HA**) or its control pCDH (**pCDH**). The interaction between SERBP1 and SIRT3 proteins was examined by immunoprecipitating with anti-HA antibody. **(G).** KMM cells transduced with SERBP1-HA (**SERBP1-HA**) or SIRT3-Myc (**SERBP1-HA**) were employed to examine the expression and colocalization of SERBP1 and SIRT3 by immunofluorescence assay (**IFA**).

Since SERBP1 deacetylation played a crucial role in KSHV-induced cellular transformation and ferroptosis, we investigated the mechanisms influencing its deacetylation. Given that deacetylation events are often regulated by deacetylases [22], we firstly determined which type of deacetylases was involved in the deacetylation of SERBP1. We evaluated the effects of deacetylase inhibitors trichostatin A (TSA, a histone deacetylase inhibitor) and nicotinamide (NAM, a sirtuins inhibitor) on SERBP1 acetylation level (Fig 3A) [26,27]. We found that NAM treatment but not TSA increased the acetylation level of SERBP1, suggesting that sirtuins were responsible for the deacetylation of SERBP1. Next, we determined the effects of SIRT1-7 on SERBP1 acetylation level. We found that SIRT3, SIRT6, and SIRT7 not only bound to SERBP1 but also decreased the acetylation levels of SERBP1 (Fig 3B). The Co-IP assay indicated that KSHV infection enhanced SIRT3 but not SIRT6 and SIRT7 binding to SERBP1 (Fig 3C and 3D). The interaction between exogenous SERBP1 and SIRT3 was further confirmed via Co-IP and immunofluorescence assays (Fig 3E–3G). These findings indicate that KSHV infection promotes SIRT3 interaction with SERBP1, resulting in a higher deacetylation state of SERBP1 in KMM cells.

**Fig 4:**
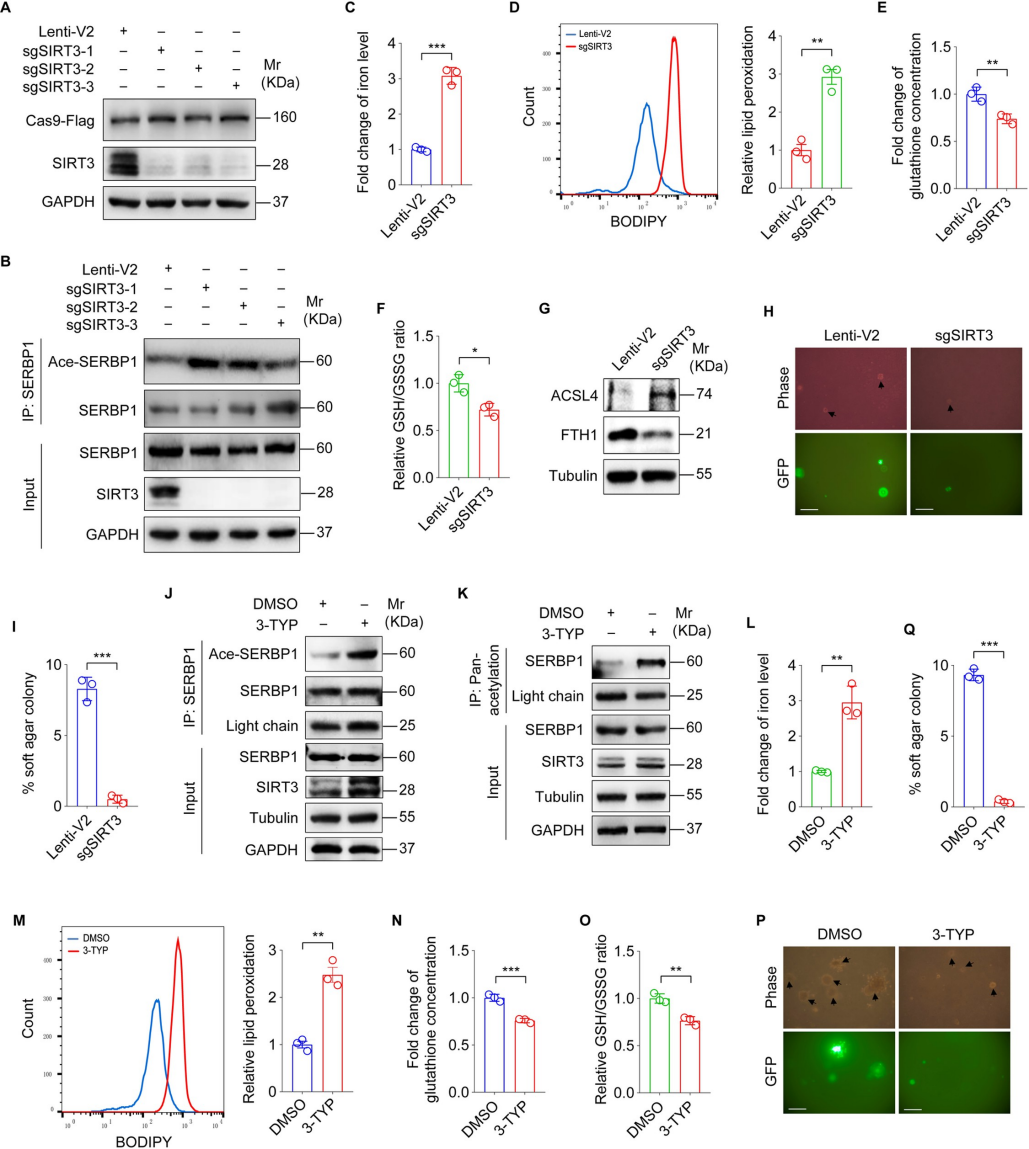
SIRT3 inhibits ferroptosis and promotes KSHV-induced cellular transformation by deacetylating SERBP1. **(A).** Western blotting analysis of SIRT3 in KMM cells infected with lentiviral sgSIRT3-1 (**sgSIRT3-1**), sgSIRT3-2 (**sgSIRT3-2**), sgSIRT3-3 (**sgSIRT3-3**), or its control lenti-V2 (**Lenti-V2**). **(B).** Cells treated as in (**A**) were utilized to examine the acetylation level of SERBP1 by immunoprecipitating with anti-SERBP1 antibody. **(C).** Levels of iron in SIRT3-knockdown KMM cells. **(D).** Flow cytometry analysis of lipid peroxidation level in SIRT3-knockdown KMM cells (**left**). The lipid peroxidation level of indicated cells was shown in bar graph (**right**). Data represented the mean ± SEM of three independent experiments. **(E).** Levels of total glutathione in SIRT3-knockdown KMM cells. **(F).** Levels of GSH/GSSG ratio in SIRT3-knockdown KMM cells. **(G).** Western blotting analysis of ACSL4 and FTH1 in SIRT3-knockdown KMM cells. **(H).** Soft agar assay of SIRT3-knockdown KMM cells. The representative images were captured at 2 weeks post seeding. Magnification, ×100. Scar bars, 40 μm. The representative images were taken from three randomly selected fields of each sample. **(I).** Quantification of the results in **(H)**. Colonies with a size equal to or larger than 20 μm (arrows shown in **H**) were counted to calculate the percentage of soft agar colonies. **(J).** KMM cells were treated with 3-TYP for 24 h, and cell lysates were immunoprecipitated with anti-SERBP1 antibody. The acetylation of SERBP1 was standardized by total SERBP1 levels. **(K).** KMM cells were treated with 3-TYP for 24 h, and cell lysates were immunoprecipitated with anti-acetylated-lysine antibody to examine SERBP1 level with anti-SERBP1 antibody by immunoblotting assay. **(L).** Levels of iron in KMM cells treated with 3-TYP or its control DMSO. **(M).** Flow cytometry analysis of lipid peroxidation level in KMM cells treated with 3-TYP or its control DMSO (**left**). The lipid peroxidation level of indicated cells was shown in bar graph (**right**). Data represented the mean ± SEM of three independent experiments. **(N).** Levels of total glutathione in KMM cells treated with 3-TYP or its control DMSO. **(O).** Levels of GSH/GSSG ratio in KMM cells treated with 3-TYP or its control DMSO. **(P).** Soft agar assay of KMM cells treated with 3-TYP or its control DMSO. The representative images were captured at 2 weeks post seeding. Magnification, ×100. Scar bars, 40 μm. The representative images were taken from three randomly selected fields of each sample. **(Q).** Quantification of the results in **(P)**. Colonies with a size equal to or larger than 20 μm (arrows shown in **P**) were counted to calculate the percentage of soft agar colonies. Data were shown as mean ± SD unless indicated else. *P < 0.05, **P < 0.01, and ***P < 0.001, Student’s *t*-test.

To further determine the regulation of SERBP1 function by SIRT3, we employed CRISPR-Cas9 method to generate SIRT3-knockdown (KD) cells (Fig 4A). We found that knockdown of SIRT3 increased the acetylation level of SERBP1 in KMM cells (Fig 4B). Furthermore, knockdown SIRT3 not only increased the iron concentration and lipid peroxidation level but also decreased glutathione concentration and GSH/GSSG ratio in KMM cells (Fig 4C–4F). Consistently, SIRT3 knockdown promoted the expression of ACSL4 and reduced the expression of FTH1 (Fig 4G). Silencing SIRT3 inhibited the efficiency of colony formation of KMM cells in soft agar (Fig 4H and 4I). 3-TYP is a selective inhibitor of SIRT3 [28]. Treatment of KMM cells with 3-TYP not only increased the acetylation level of SERBP1 (Fig 4J and 4K) but also elevated the iron concentration and lipid peroxidation level (Fig 4L and 4M), and lowered the glutathione concentration and GSH/GSSG ratio (Fig 4N and 4O). Consistently, 3-TYP treatment suppressed the efficiency of colony formation of KMM cells in soft agar (Fig 4P and 4Q).

To determine whether SIRT3 inhibited ferroptosis by inducing SERBP1 deacetylation, we co-expressed SIRT3 and SERBP1-K68R in SERBP1 KO KMM cells. We found no discernible change in the level of SERBP1 deacetylation, suggesting that K68R mutation of SERBP1 effectively abolishes SIRT3-mediated deacetylation (S3A Fig). Meanwhile, overexpression of SIRT3 together with SERBP1-K68R in SERBP1 KO KMM cells did not change the iron concentration, lipid peroxidation level, the glutathione concentration and GSH/GSSG ratio compared to the pCDH group (S3B–S3E Fig).

Taken together, these results suggest that SIRT3 promotes the deacetylation of SERBP1 at lysine K68, thereby enhancing KSHV-induced cellular transformation by inhibiting ferroptosis.

### vIL-6 hijacks SIRT3 to promote SERBP1 deacetylation, leading to the inhibition of ferroptosis

**Fig 5:**
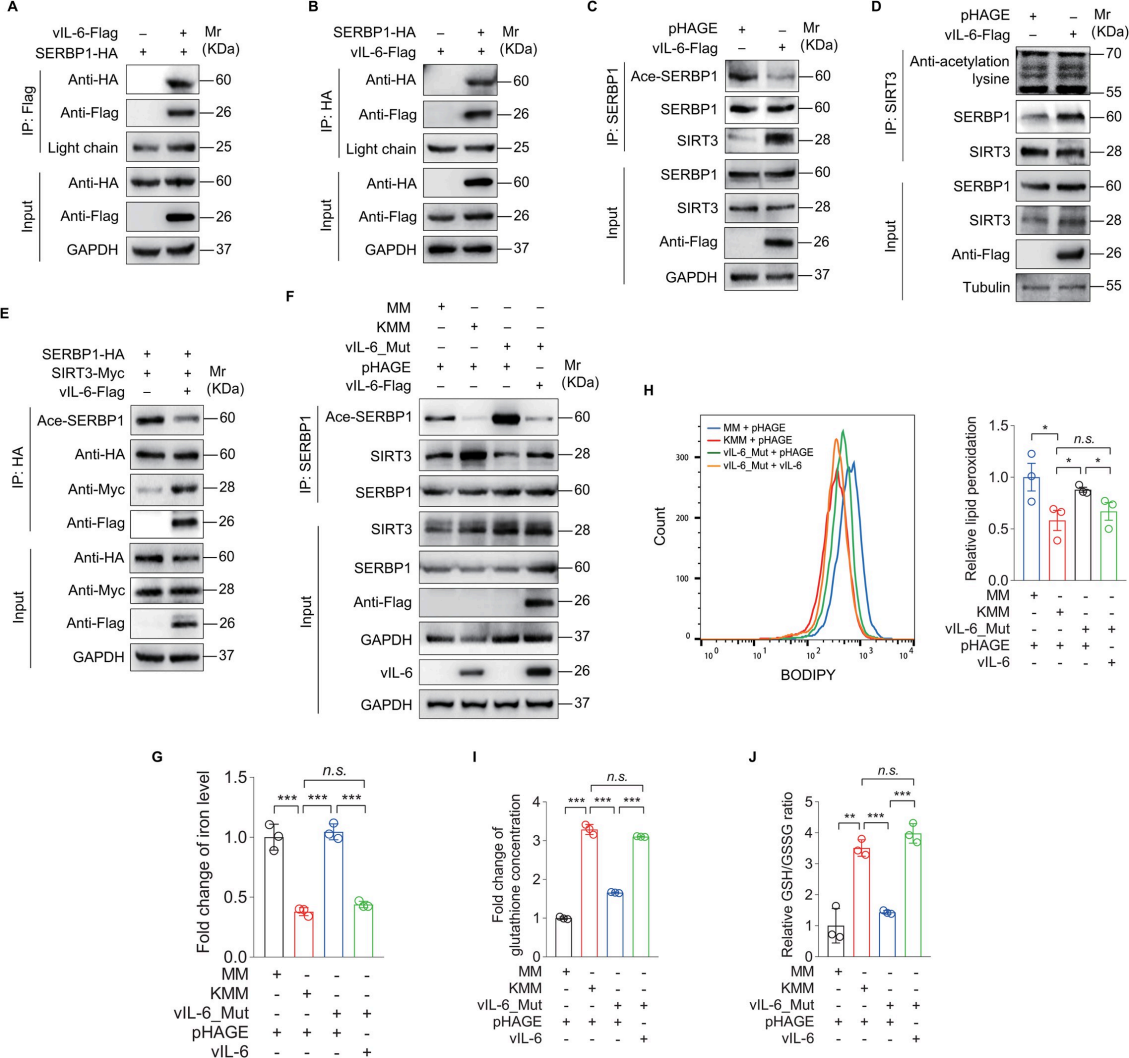
KSHV vIL-6 hijacks SIRT3 to promote the deacetylation of SERBP1 which inhibits ferroptosis. **(A).** KMM cells infected with lentiviral SERBP1-HA (**SERBP1-HA**) were transduced with lentiviral vIL-6-Flag (**vIL-6-Flag**) or its control pHAGE. The interaction between vIL-6 and SERBP1 proteins was examined by immunoprecipitating with anti-Flag antibody. **(B).** KMM cells infected with vIL-6-Flag (**vIL-6-Flag**) were transduced with lentiviral SERBP1-HA (**SERBP1-HA**) or its control pCDH. The interaction between SERBP1 and vIL-6 proteins was examined by immunoprecipitating with anti-HA antibody. **(C).** KMM cells were transduced with lentiviral vIL-6-Flag (**vIL-6-Flag**) or its control pHAGE (**pHAGE**). The acetylation levels of SERBP1 in these cells were examined by immunoprecipitating with anti-SERBP1 antibody. **(D).** KMM cells were transduced with lentiviral vIL-6-Flag (**vIL-6-Flag**) or its control pHAGE (**pHAGE**). The interaction between SIRT3 and SERBP1 proteins was examined by immunoprecipitating with anti-SIRT3 antibody. The acetylation proteins interacted with SIRT3 were examined with anti-acetylation lysine antibody. **(E).** KMM cells co-infected with lentiviral SERBP1-HA (**SERBP1-HA**) and SIRT3-Myc (**SIRT3-Myc**) were transduced with lentiviral vIL-6-Flag (**vIL-6-Flag**) or its control pHAGE. The interaction between SIRT3 and SERBP1 proteins was examined by immunoprecipitating with anti-HA antibody. The acetylation level of SERBP1 was examined by immunoblotting with anti-acetylated-lysine antibody. **(F).** MM cells infected by wild type KSHV (**KMM**) (MOI of 3) or a mutant virus with a deletion of KSHV ORF-K2 (**K2_Mut;** ORF-K2 encoding vIL-6) (MOI of 3) for 6 h were transduced with lentiviral vIL-6-Flag (**vIL-6-Flag**) for 48 h. Cells were collected and immunoprecipitated with anti-SERBP1 antibody. The acetylation level of SERBP1 was standardized by total SERBP1 levels. **(G).** Levels of iron of cells treated as in **(F). (H).** Flow cytometry analysis of the lipid peroxidation level in cells treated as in **(F)** (**left**). The lipid peroxidation level of indicated cells was shown in bar graph (**right**). Data represented the mean ± SEM of three independent experiments. **(I).** Levels of total glutathione of cells treated as in **(F)**. **(J).** Levels of GSH/GSSG ratio of cells treated as in **(F)**. Data were shown as mean ± SD unless indicated else. *P < 0.05, **P < 0.01, and ***P < 0.001, Student’s *t*-test. *n*.*s*., not significant.

Because SERBP1 had stronger binding to SIRT3 in KMM than MM cells, we hypothesized that a KSHV-encoded protein might promote SIRT3 interaction with SERBP1. We examined the interaction of SERBP1 with KSHV-encoded latent and early lytic proteins by performing Co-IP. We found that SERBP1 bound to KSHV vIL-6, but not vFLIP, vCyclin and LANA (Figs 5A and 5B and S4A–S4C). Overexpression of vIL-6 increased SIRT3 binding to SERBP1, which was accompanied by a decrease in SERBP1 acetylation level (Fig 5C and 5D). Similar results were observed following co-expression of SERBP1 and SIRT3 proteins in the presence of vIL-6 (Fig 5E). While the level of SERBP1 acetylation was decreased in KMM cells, deletion of vIL-6 (vIL-6_Mut) restored the level of SERBP1 acetylation (Fig 5F). Furthermore, complementation with vIL-6 in the vIL-6 deletion cells completely reversed these effects (Fig 5F).

Our previous study has reported that deletion of vIL-6 abolishes colony formation in soft agar of KSHV-transformed cells [23]. To determine whether ferroptosis is involved in this process, we examined ferroptosis-related indicators. We showed an enhanced ferroptosis in vIL-6_Mut cells, which was reversed by the supplementation with vIL-6 (Fig 5G–5J). Together these results suggest that vIL-6 promotes SIRT3 binding to SERBP1, which increases the SERBP1 deacetylation level, resulting in inhibition of ferroptosis and enhancement of KSHV-induced cellular transformation.

### Deacetylated SERBP1 binds less efficiently to Lipt2 mRNA, causing its instability and a lower expression

**Fig 6:**
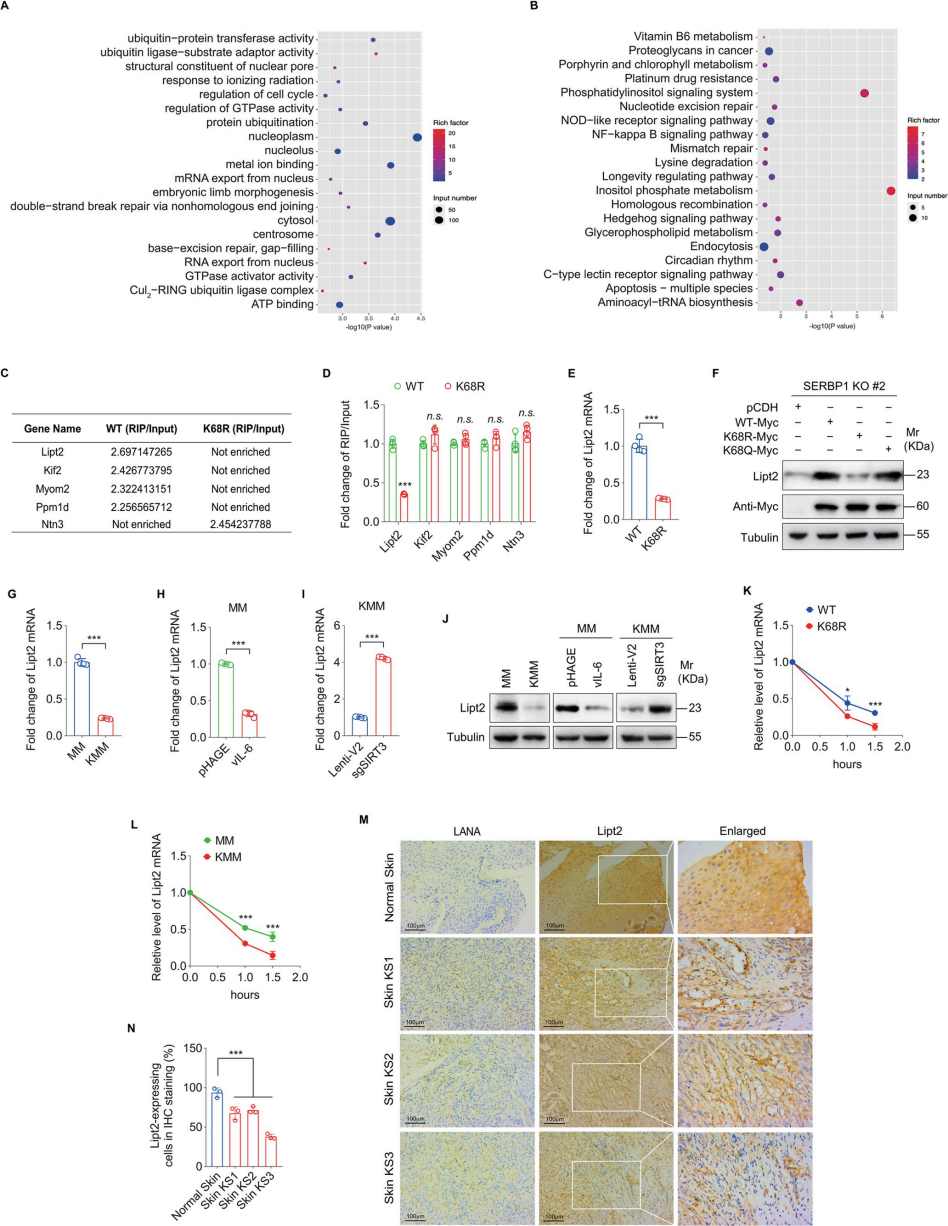
Deacetylated SERBP1 interacts with less Lipt2 mRNA and increases its instability. **(A).** GO enrichment analyses of SERBP1 knockout (**KO**) KMM cells transduced with lentiviral wild type SERBP1 (**WT**) or K68R mutant SERBP1 (**K68R**). **(B).** KEGG pathway of SERBP1 knockout (**KO**) KMM cells transduced with lentiviral wild type SERBP1 (**WT**) or K68R mutant SERBP1 (**K68R**). **(C).** Differentially interacted mRNAs obtained in 3 paired samples from SERBP1 knockout (**KO**) KMM cells transduced with lentiviral wild type SERBP1 (**WT**) or K68R mutant SERBP1 (**K68R**). **(D).** RNA immunoprecipitation followed by qPCR analysis of the interaction between SERBP1 and differentially interacted mRNAs in SERBP1 knockout (KO) KMM cells transduced with lentiviral wild type SERBP1 (**WT**) or K68R mutant SERBP1 (**K68R**). GAPDH was used as a control. **(E).** RT-qPCR analysis of Lipt2 mRNA level in SERBP1 knockout (KO) KMM cells transduced with lentiviral wild type SERBP1 (**WT**) or K68R mutant SERBP1 (**K68R**). **(F).** Western blotting analysis of Lipt2 expression in SERBP1 knockout (KO) KMM cells transduced with lentiviral wild type SERBP1 (**WT-Myc**), K68R mutant SERBP1 (**K68R-Myc**), or K68Q mutant SERBP1 (**K68Q-Myc**). **(G).** RT-qPCR analysis of Lipt2 mRNA level in MM and KMM cells. **(H).** RT-qPCR analysis of Lipt2 mRNA level in MM cells transduced with lentiviral vIL-6 or its control pHAGE. **(I).** RT-qPCR analysis of Lipt2 mRNA level in KMM cells infected with lentiviral sgSIRT3 (**sgSIRT3**) or its control lenti-V2 (**Lenti-V2**). **(J).** Western blotting analysis of Lipt2 expression of cells treated as in (**G**), (**H**), and (**I**), respectively. **(K).** SERBP1 knockout (KO) KMM cells transduced with lentiviral wild type SERBP1 (**WT**) or K68R mutant SERBP1 (**K68R**) were treated with actinomycin D. RNA decay assay was performed to detect the degradation rate of Lipt2 mRNA. **(L).** MM or KMM cells were treated with actinomycin D. RNA decay assay was performed to examine the degradation rate of Lipt2 mRNA. **(M).** Immunohistochemical staining (IHC) of Lipt2 in normal skin, skin KS of patient #1 (**Skin KS1**), skin KS of patient #2 (**Skin KS2**), and skin KS of patient #3 (**Skin KS3**). Magnification, ×200, ×400. **(N).** Quantification of the results in (**M**). Data were shown as mean ± SD unless indicated else. *P < 0.05 and ***P < 0.001, Student’s *t*-test. *n*.*s*., not significant.

As an RNA-binding protein, SERBP1 plays multiple functions in mRNA metabolism including regulation of mRNA stability and translation [29–33]. To identify the mRNAs influenced by SERBP1 acetylation, RNA immunoprecipitation coupled to RNA sequencing (RIP-Seq) was performed on SERBP1 knockout (KO) KMM cells overexpressing wild type SERBP1 (SERBP1-WT) or K68R mutant of SERBP1 (SERBP1-K68R). GO and KEGG analyses showed that genes of SERBP1 binding mRNAs were involved in protein ubiquitination, cell cycle, mRNA export and other biological processes (Fig 6A and 6B). We chose the top five genes whose mRNAs were most differentially bound by SERBP1-WT and SERBP1-K68R for the validation experiments (Fig 6C). RIP-qPCR revealed that SERBP1 K68R mutant bound to Lipt2 mRNA much less efficiently than that of SERBP1-WT (Fig 6D). The mRNA level of Lipt2 was also lower in SERBP1 KO KMM cells expressing the SERBP1 K68R mutant than SERBP1-WT (Fig 6E). Western blotting confirmed a decrease in Lipt2 protein level in SERBP1 K68R mutant cells and an increase in SERBP1 K68Q mutant cells compared to SERBP1-WT cells (Fig 6F). Furthermore, a significant decrease in Lipt2 mRNA level were observed in KMM cells (Fig 6G), as well as in MM cells overexpressing vIL-6 (Fig H). Knockdown SIRT3 elevated Lipt2 mRNA level in KMM cells (Fig I). These results were further confirmed at the protein level (Fig 6J). RNA stability experiments indicated that overexpressing SERBP1-K68R in SERBP1 KO KMM cells resulted in the promotion of Lipt2 mRNA degradation (Fig 6K). A significant decrease of Lipt2 mRNA stability was also observed in KMM cells (Fig 6L). Importantly, Lipt2 proteins in KS lesions were downregulated compared to normal skin tissues (Fig 6M and 6N). Collectively, these results reveal that deacetylation of SERBP1 at K68 prevents its binding to Lipt2 mRNA, which induces its instability, leading to lower levels of its mRNA and protein in KMM cells and KS tumor cells.

### Overexpression of Lipt2 promotes ferroptosis and inhibits KSHV-induced cellular transformation

**Fig 7:**
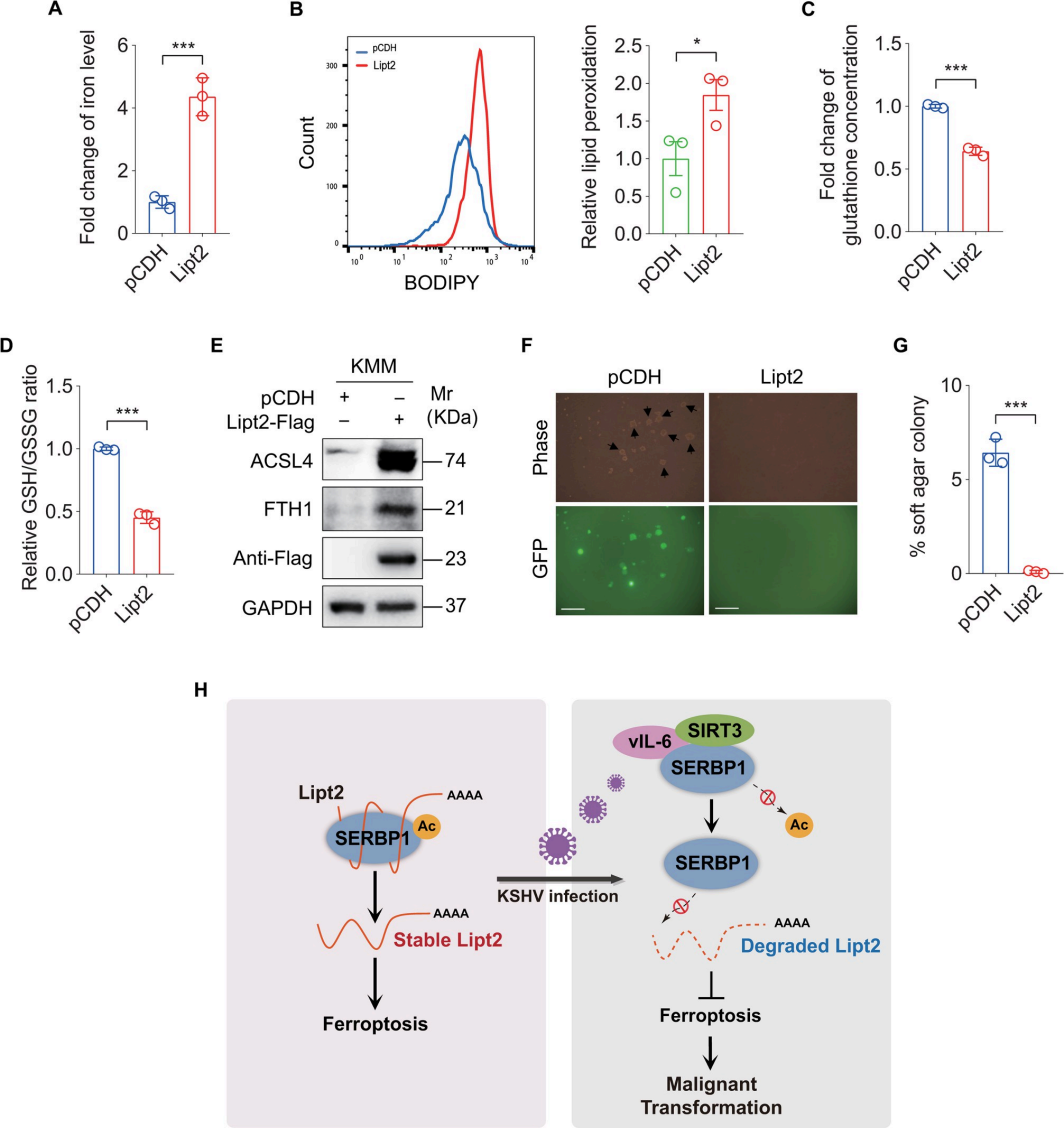
Overexpression of Lipt2 promotes ferroptosis and inhibits KSHV-induced cellular transformation. **(A).** Levels of iron in KMM cells transduced with lentiviral Lipt2-Flag (**Lipt2-Flag**) and its control pCDH (**pCDH**). **(B).** Flow cytometry analysis of lipid peroxidation level in cells treated as in (**A**) (**left**). The lipid peroxidation level of indicated cells was shown in bar graph (**right**). Data represented the mean ± SEM of three independent experiments. **(C).** Levels of total glutathione in cells treated as in (**A**). **(D).** Levels of GSH/GSSG ratio in cells treated as in (**A**). **(E).** Western blotting analysis of ACSL4 and FTH1 in cells treated as in (**A**). **(F).** Soft agar assay of cells treated as in (**A**). The representative images were captured at 2 weeks post seeding. Magnification, ×100. Scar bars, 40 μm. The representative images were taken from three randomly selected fields of each sample. **(G).** Quantification of the results in **(F)**. Colonies with a size equal to or larger than 20 μm (arrows shown in **F**) were counted to calculate the percentage of soft agar colonies. **(H).** A schematic working model of the mechanism by which SERBP1 deacetylation contributes to KSHV-induced cellular transformation by inhibiting ferroptosis. Data were shown as mean ± SD unless indicated else. *P < 0.05 and ***P < 0.001, Student’s *t*-test.

To determine the role of Lipt2 in KSHV-induced cellular transformation, we overexpressed Lipt2 in KMM cells. We found a significant increase in cellular iron and lipid peroxidation level (Fig 7A and 7B), and a dramatic decrease in glutathione level and GSH/GSSG ratio (Fig 7C and 7D), as well as an increase level of ACSL4 (Fig 7E). However, the expression of FTH1 was unexpectedly enhanced (Fig 7E). Importantly, overexpression of Lipt2 inhibited the colony formation ability of KMM cells (Fig 7F and 7G). Together these results indicate that downregulation of Lipt2 expression mediated by SERBP1 deacetylation in KMM cells inhibits ferroptosis and promotes KSHV-induced cellular transformation.

## Discussion

Recently, emerging studies focusing on ferroptosis have shed light on its involvement in the development and therapeutic responses of various types of tumors [13,34–38]. In fact, ferroptosis has a dual role in viral infection and cancer [39]. On the one hand, ferroptosis is a host defensive strategy against pathogenic infections. Thus, pathogens must evolve mechanisms to inhibit ferroptosis during infections [38]. While some pathogens are required to induce cell death to release progeny [40,41], others, particularly viruses undergo latent infections, have evolved to inhibit cell death to promote cell proliferation or evade host immune defenses [42]. On the other hand, during tumorigenesis, the functions of ferroptosis may depend on the release of damage-associated molecular patterns and the activation of immune responses triggered by ferroptotic damage within the tumor microenvironment [13,15]. As an important regulator of ferroptosis, the cystine/glutamate antiporter SLC7A11/xCT functions to import cystine for glutathione biosynthesis and antioxidant defense and is overexpressed in multiple human cancers [43–45]. It has been reported that upregulation of SLC7A11 by KSHV-encoded miR-K12-11 facilitates KSHV dissemination and *de novo* infection. Targeting SLC7A11 induces apoptosis and tumor regression for KSHV/HIV-associated lymphoma [46–48]. High expression of SLC7A11 renders otherwise unsusceptible target cells permissive for both KSHV cell fusion and virion entry [49,50]. These reports involving SLC7A11 function imply that ferroptosis may participate in KSHV infection and tumorigenesis. In this study, for the first time to our knowledge, we have discovered that ferroptosis is significantly inhibited in KSHV-transformed cells and revealed that KSHV vIL-6 mediates KSHV-induced ferroptosis inhibition. These findings uncovered an important mechanism of inhibiting ferroptosis in KSHV-induced tumorigenesis. Remarkably, we did not detect any changes in the expression level of SLC7A11 in KMM cells, which contradicts the previously reported upregulation of SLC7A11 in KS lesions or KSHV-infected PEL cells [46–49]. This is possible that this discrepancy is due to the difference of cell types involved in these studies.

In addition to SLC7A11, there are several other key modulators of the ferroptosis, including glutathione peroxidase 4 (GPX4), ACSL4 and FTH1. GPX4 is a selenoenzyme that depends on glutathione (GSH) and plays a crucial role in preventing ferroptosis by reducing toxic lipid hydroperoxides to nontoxic lipid alcohols [51]. ACSL4 is essential for iron-dependent cellular death, and ferroptosis is executed through the production of reactive oxygen species (ROS) by ACSL4 [52]. FTH1, which interacts with nuclear receptor coactivator 4 (NCOA4), facilitates the delivery of iron-bound ferritin to autophagosomes for lysosomal degradation and subsequent iron release. Consequently, FTH1 acts as a negative regulator of ferroptosis [53]. In this study, we observed the downregulation of ACSL4 and the upregulation of FTH1 in KMM cells, while GPX4 expression remained unchanged. These findings suggest that KSHV-induced ferroptosis inhibition likely occurs through the regulation of ACSL4 and FTH1 expression. Furthermore, SERBP1 deacetylation and SIRT3 were also found to regulate ACSL4 and FTH1 expression. Surprisingly, overexpression of Lipt2 resulted in an increased level of ACSL4 but unexpectedly enhanced FTH1 expression. These results imply that SERBP1 deacetylation-mediated inhibition of ferroptosis may involve other regulators of ferroptosis in addition to FTH1.

Serpine mRNA binding protein 1 (SERBP1) has been identified as a binding protein to the 3’ region of the mRNA encoding plasminogen activator inhibitor (PAI) type I, regulating PAI mRNA stability [54]. Increased level of SERBP1 has been observed in various cancers, including acute lymphoblastic leukemia, breast cancer, ovarian carcinoma, glioblastoma and squamous lung-cell carcinoma. In addition, various human cancer cell lines that develop resistance to cisplatin also expressed increased level of SERBP1 [55–61]. Although SERBP1 is known to be an RNA binding protein, little is known about its RNA targets. Previous studies have demonstrated that SERBP1 affects homologous recombination-mediated DNA repair in response to DNA double-strand breaks by regulating CtIP translation in S phase [31]. Meanwhile, SERBP1 is asymmetrically dimethylated by PRMT1 and this modification affects its protein interaction and intracellular localization of the protein [32]. Except for dimethylation, study on the post-translation modification (PTM) of SERBP1 is almost nonexistent. Here, we have revealed that KSHV infection does not influence SERBP1 protein expression, but increases the SERBP1 deacetylation level. Importantly, SERBP1 deacetylation inhibits ferroptosis and promotes KSHV-induced cellular transformation. However, whether there are other novel acetylated modifications on SERBP1 and whether these modifications are associated with ferroptosis and KSHV-induced cellular transformation require further investigations.

Sirtuin 3 (SIRT3) is a member of the mammalian sirtuin family and is involved in the regulation of multiple cellular processes [62–65]. As a major mitochondrial NAD+-dependent deacetylase [66,67], SIRT3 target diverse substrate proteins to perform various functions including regulation of oxidative stress, reprogramming of tumor cell energy pathways, and metabolic homeostasis [62–67]. In the current study, we revealed that SIRT3 mediates KSHV-induced cellular transformation by interacting with SERBP1 and deacetylating SERBP1. Knockdown of SITR3 or inhibition of SIRT3 activity with its chemical inhibitor 3-TYP is sufficient to decrease the ability of KSHV-induced cellular transformation. Therefore, targeting SIRT3 to block the deacetylation of SERBP1 represents an attractive therapeutic strategy in KSHV-related malignancies.

Viral interleukin-6 (vIL-6) is a homolog of human IL-6, it could directly bind to the gp130 subunit of IL-6 receptor to induce STAT3 phosphorylation by activating the JAK/STAT pathway [68]. Although vIL-6 is an early viral lytic gene, it is widely expressed in KS spindle cells and in MCD, indicating its critical role in KSHV tumorigenesis. Our previous study showed that vIL-6 increases the acetylation of STAT3 to promote DNA methyltransferase 1 (DNMT1)-mediated epigenetic silencing of CAV1 [59]. Here, we have found that, besides STAT3 acetylation, vIL-6 also regulates SERBP1 deacetylation. vIL-6 facilitates SIRT3 binding to SERBP1, leading to increased SERBP1 deacetylation, inhibition of ferroptosis, promotion of cellular transformation.

Lipoyltransferase 2 (Lipt2) is a mitochondrial protein that catalyzes the transfer of octanoic acid to lipoate-dependent enzymes. Defects of lipoic acid synthesis and transfer as a result of Lipt2 mutation have been identified as causes of several diseases [69,70]. However, the role of Lipt2 in KSHV infection and KS pathogenesis has not been characterized before. In this study, we have observed that Lipt2 promotes ferroptosis. KSHV vIL-6 induced SERBP1 deacetylation prevents its binding to Lipt2 mRNA, induces its mRNA degradation resulting in lower protein expression. Furthermore, overexpression of Lipt2 in KSHV-transformed cells is sufficient to promote ferroptosis and abolish the colony formation of KSHV-transformed cells. This is the first time that a role of Lipt2 in ferroptosis and KS pathogenesis has been identified. However, how Lipt2 regulates ferroptosis to contribute to KSHV-induced tumorigenesis requires further investigations.

In summary, our study reveals an essential role of SERBP1 deacetylation in KSHV-induced cellular transformation. vIL-6 promotes SIRT3 binding to SERBP1, causing the deacetylation of SERBP1. Deacetylated SERBP1 exhibits reduced binding to Lipt2 mRNA, inducing Lipt2 mRNA instability, which inhibits ferroptosis and promotes KSHV-induced tumorigenesis (Fig 7H). Our findings unveil a novel role of SERBP1 in KSHV-induced cellular transformation and identify a potential therapeutic target for KSHV-induced cancers.

## Materials and methods

### Ethics statement

A total of three pairs of KS patients’ lesions and normal skin tissues were collected from Changzhou Third People’s Hospital. The Institutional Ethics Committee of Changzhou Third People’s Hospital reviewed and ethically approved this study. Written informed consent was obtained from all participants, and all samples were anonymized.

### Cell culture and transfection

HEK293T and primary rat embryonic metanephric mesenchymal precursor (MM) cells were cultured in Dulbecco’s modified Eagle’s medium (DMEM) containing 10% fetal bovine serum (FBS). KSHV-transformed MM (KMM) cells were maintained in DMEM supplemented with 10% FBS and 150 μg/ml hygromycin B [25]. The hygromycin B selection was removed one week prior to experiments. HEK293T cells, used for lentivirus packaging were transfected with Lipofectamine 2000 Reagent (Invitrogen, Carlsbad, CA, USA). All cells used in this study were examined for mycoplasma contamination using Myco-Blue Mycoplasma Detector (D103, Vazyme Biotech Co., Ltd, Nanjing, China).

### Reagents and plasmids

3-TYP, a selective inhibitor of SIRT3, was obtained from Selleck Chemicals (Shanghai, China). Actinomycin D was purchased from APE-Bio (Houston, USA). TSA and NAM were from MedChemExpress LLC (Shanghai, China). The expressing plasmids of vIL-6-Flag and vFLIP-Flag were generated as previously described [24,59]. The skeleton plasmid for lentiviral vectors vCyclin-Myc, SERBP1-HA/Myc, SIRTs-Myc and Lipt2-Flag was pCDH-CMV-MCS-EF1-copGFP (referred to as pCDH). The expressing plasmid of LANA-Flag was kindly provided by Dr. Ke Lan from Wuhan University. The small guide RNA sequences (sgRNAs) targeting SERBP1 and SIRT3 are listed in S1 Table. The control vector of all sgRNAs was Lenti-CRISPR-V2 (Lenti-V2 for short). All constructs were verified through DNA sequencing.

### Lentivirus packaging and infection

HEK293T cells were co-transfected with lentiviral plasmids, packaging plasmid psPAX2, and envelope plasmid pMD2.G using Lipofectamine 2000 Reagent (Invitrogen, Carlsbad, CA, USA) following the previously described protocol [71–73].

### Tandem mass tag (TMT) labeling proteomic technique

Quantitative proteomics profiling of lysine acetylation was performed by Shanghai Applied protein technology Co., Ltd. Briefly, the sample was dissolved in lysis buffer. The supernatant was collected after centrifugated at 15,000 g for 30 min at 4°C, and the protein concentrations were quantified by Bradfford method. Then, the sample was digested in trypsin, lyophilized, and re-dissolved with 100 mM dissolution buffer for labeling. The TMT reagent was added to digested sample. Subsequently, the labeled samples were incubated with Anti-Ac-K antibody beads [PTMScan Acetyl-Lysine Motif (Ac-K) kit, Cell Signal Technology] for enrichment of acetylated peptides and then analyzed by Easy nLC system coupled with Q-Exactive mass spectrometer. Raw LC–MS/MS data were processed by MaxQuant software (v1.5.3.17). Data were searched against the UniProt Rat complete proteome database of reviewed (Swiss-Prot) and unreviewed (TrEMBL) proteins. From the 2480 AcK-peptides identified, peptides passed the threshold for an adjusted *P* value ≤ 0.05. Functional enrichment analysis was performed by using KAAS software tool (KEGG Automatic Annotation Server).

### Colony formation assay in soft agar

The soft agar assay was performed as previously described [23]. Agar was used as the culture medium to allow cells to grow in a suspended state. a suspension of 2x10^4^ cells in 2 mL of 0.3% top agar (BD Biosciences) was plated onto 2 mL 0.6% base agar in a well of 6 well-plates. The upper layer of soft agar disperses cells into individual cells, while the lower layer of soft agar serves as a support to prevent cell adhesion and growth. After 2 weeks, we selected three fields randomly of each sample and photographed the images. Colonies with a size equal to or larger than 20 μm were counted to calculate the percentage of soft agar colonies. The representative images were taken from three randomly selected fields of each sample.

### RNA extraction and RT-qPCR

According to the manufacturer’s instruction, total RNA was extracted from the cells using TRIzol reagent (Invitrogen, USA). cDNAs were generated with HiScript III RT SuperMix for qPCR (Vazyme Biotech Co., China Nanjing, China). Real-time quantitative PCR was conducted with ChamQ SYBR qPCR Master Mix (Vazyme Biotech Co., Ltd, Nanjing, China) on Applied Biosystems (ABI, Foster City, CA, USA). Primer sequences are listed in S2 Table. GAPDH was utilized as an internal normalization control.

### Western blotting

Western blotting analysis was performed as described previously [74]. Acetylated-lysine antibody was obtained from Cell Signaling Technologies (Danvers, USA). Anti-SIRT3 rabbit antibody, anti-SIRT6 rabbit antibody, anti-SIRT7 rabbit antibody, and anti-ACSL4 mouse antibody were from Proteintech Group, Inc. (Wuhan, China). Anti-Lipt2 rabbit antibody was obtained from Bioss (Beijing, China). Anti-FTH1 rabbit antibody was obtained from Abclonal (Wuhan, China). Anti-GAPDH mouse antibody was from Santa Cruz Biotechnology (Dallas, TX, USA). Anti-Myc mouse antibody, anti-HA mouse antibody and anti-Flag mouse antibody were purchased from MEDICAL & BIOLOGICAL LABORATORIES CO., LTD. (Tokyo, Japan). Anti-vIL-6 rabbit monoclonal antibody was kindly provided by Dr Robert Yarchoan from Center for Cancer Research, National Cancer Institute (Bethesda, Maryland, USA).

### Co-immunoprecipitation

Cells were lysed in 500 μL of lysis buffer solution (Thermo fisher, USA) supplemented with protease inhibitor and phosphatase inhibitor (bimake, China). The supernatants of the cell lysates were obtained by centrifugation at 13,000 rpm for 10 min at 4°C. 50 μL of cell lysates supernatants were reserved as whole cell lysate. The remaining supernatants were incubated with specified magnetic beads at 4°C overnight. After washing three times with TBST (10mM Tris-HCl, 150mM NaCl, 0.1%Tween-20), SDS loading buffer was added and the samples were boiled for 5 min prior to Western blotting analysis.

### RNA immunoprecipitation (RIP) and RIP-seq

RIP assay was performed using Magna RIP Kit (17–701, Millipore) according to the instructions by the manufacturer. Briefly, 5 μg of anti-Myc (MEDICAL & BIOLOGICAL LABORATORIES CO., LTD.) or anti-Mouse IgG (Millipore) was incubated with 50 μL of magnetic beads before cell lysates were added (approximately 2 × 10^7^ cells per sample). Subsequently, RNAs were extracted after the removal of proteins and purified for qPCR analysis. The relative enrichment was normalized to the input as follows: %Input = 2^^-(Ct [IP]–(Ct [input]-LOG2[10])^. The RIP-seq process and analysis were performed by SEQHEALTH Co., Ltd. (Wuhan, China). The raw data could be found in GEO (GEO accession number GSE241111).

### Measurement of iron level

Cells were harvested and homogenized in 4–10 volumes of Iron Assay Buffer using a Dounce homogenizer sitting at 4°C. Then iron level in the cells was measured using kits from Abcam (ab83366) according to the manufacturer’s instructions.

### Measurement of lipid peroxidation

The fluorescent probe BODIPY-C11 (665/676) (Invitrogen, Waltham, MA, USA) was used to detect lipid peroxides by flow cytometry following a previously described [75]. Briefly, cells were treated with 500 nM BODIPY-C11 (665/676) for 30 min at 37°C. After incubation, cells were washing twice with PBS, trypsinized, and analyzed for fluorescence of the oxidized form of BODIPY-C11 using BD FACSCalibur, CellQuest Pro (BD). Flowjo V10.8.0 software was used for data analysis.

### Measurement of GSH and GSSG

GSH and GSSG Assay Kit (Beyotime, Shanghai, China) was used to measure the GSH and GSSG levels according to the manufacturer’s instructions. The GSH levels were quantified colorimetrically by measuring the absorbance of each well at a wavelength of 412 nm (OD412). The calculations were performed based on the standard curve.

### mRNA stability assay

For mRNA stability evaluation, cells were seeded into 12-well plates and treated with actinomycin D (5 μg/mL) (APE-Bio, USA) for 0, 0.5, 1.0, 1.5, and 2 h. Following the respective treatment times, cells were lysed in TRIzol reagent (Invitrogen, USA) for RNA extraction and RT-qPCR was performed to measure mRNA levels.

### Immunofluorescence assay (IFA)

Cells were fixed with cold acetone for 10 min. After washing, cells were incubated in PBS containing 0.3% Triton X-100 for 10 min and blocked with 1% BSA for 30 min. Then, cells were incubated with anti-Myc antibody (MEDICAL & BIOLOGICAL LABORATORIES CO., LTD., mouse, 1:500 dilution), and anti-HA antibody (Proteintech, rabbit, 1:100 dilution) at 4°C overnight. Following additional washes, cells were incubated with the appropriate secondary antibody (1:200; Beyotime Biotechnology) at room temperature for 1 h, and then stained with DAPI. Images were acquired using Zeiss Laser scanning confocal microscope (Lsm880 NLO).

### Immunohistochemistry (IHC)

For the IHC analysis, anti-KSHV LANA antibody (Abcam, UK) and anti-Lipt2 rabbit antibody (Bioss, China) were employed. The visualization of the staining was performed using the DAB (3,3′-diaminobenzidine) Peroxidase (HRP) Substrate Kit (Vector Laboratories, Inc., Burlingame, USA).

### Statistical analysis

All experiments were independently with a minimum of three replicates with similar results, and results are presented as the mean ± SD from at least 3 technical replicates or mean ± SEM from 3 biological replicates as indicated in the figure legends. Statistical analysis was based on chi-square test for Fig 6N. The remaining statistical comparisons between two groups were done using unpaired Student’s *t-*test. *P* values of less than 0.05 were considered significant.

## Supporting information

S1 Fig Mass spectrometry analysis of acetylation sites of SERBP1. Acetylation of SERBP1 lysine residues at Lys68, 102, 140, 278, 304, 374 were identified in both MM and KMM cells by tandem mass tag (TMT) labeling proteomic technique. The b and y ions in the spectra of the peptide were marked in green and orange, respectively.
(TIF)

S2 Fig The colony formation ability analysis of KMM cells treated with ferroptosis inhibitor. **(A).** Soft agar assay of KMM cells treated with 10 μM Fer-1 for 24h. The representative images were captured at 2 weeks post seeding. Magnification, ×100. Scar bars, 40 μm. The representative images were taken from three randomly selected fields of each sample. **(B).** Quantification of the results in (**A)**. Colonies with a size equal to or larger than 20 μm (arrows shown in **A**) were counted to calculate the percentage of soft agar colonies.
(TIF)

S3 Fig SIRT3 inhibits ferroptosis by inducing SERBP1 deacetylation at lysine K68. **(A).** SERBP1 KO KMM cells infected with lentiviral SERBP1 WT-HA (**WT-HA**) or SERBP1 K68R-HA (**K68R-HA**) were transduced with lentiviral SIRT3-Myc (**SIRT3-Myc**) or its control pCDH (**pCDH**). The acetylation of SERBP1 was examined by immunoprecipitating with anti-HA antibody. **(B).** Levels of iron in cells treated as in (**A**). **(C)**. Flow cytometry analysis of lipid peroxidation level in cells treated as in (**A**) (**left**). The lipid peroxidation level of indicated cells was shown in bar graph (**right**). **(D)**. Levels of total glutathione in cells treated as in (**A**). **(E)**. Levels of GSH/GSSG ratio in cells treated as in (**A**). Data were shown as mean ± SD. **P < 0.01 and ***P < 0.001, Student’s *t*-test. *n*.*s*., not significant.
(TIF)

S4 Fig The interaction between SERBP1 and partial latency genes-encoded proteins of KSHV. **(A)**. KMM cells infected with lentiviral SERBP1-HA (**SERBP1-HA**) were transduced with lentiviral vFLIP-Flag (**vFLIP-Flag**) or its control pHAGE. The interaction between vFLIP and SERBP1 proteins was examined by immunoprecipitating with anti-Flag antibody. **(B)**. KMM cells infected with lentiviral SERBP1-HA (**SERBP1-HA**) were transduced with lentiviral vCyclin-Myc (**vCyclin-Myc**) or its control pCDH. The interaction between vCyclin and SERBP1 proteins was examined by immunoprecipitating with anti-Myc antibody. **(C)**. KMM cells infected with lentiviral SERBP1-HA (**SERBP1-HA**) were transduced with lentiviral LANA-Flag (**LANA-Flag**) or its control pCDH. The interaction between LANA and SERBP1 proteins was examined by immunoprecipitating with anti-Flag antibody.
(TIF)

S1 Table The sequences of the sgRNAs. (PDF)

S2 Table The primers used in this study. (PDF)

S1 Data Uncropped images. (PDF)
