# Supplementary figures and images for: KSHV vIL-6 promotes SIRT3-induced deacetylation of SERBP1 to inhibit ferroptosis and enhance cellular transformation by inducing lipoyltransferase 2 mRNA degradation
Source: PLoS Pathog. 2024 Mar 12;20(3):e1012082. doi: 10.1371/journal.ppat.1012082 (PMC10959363; doi:10.1371/journal.ppat.1012082)

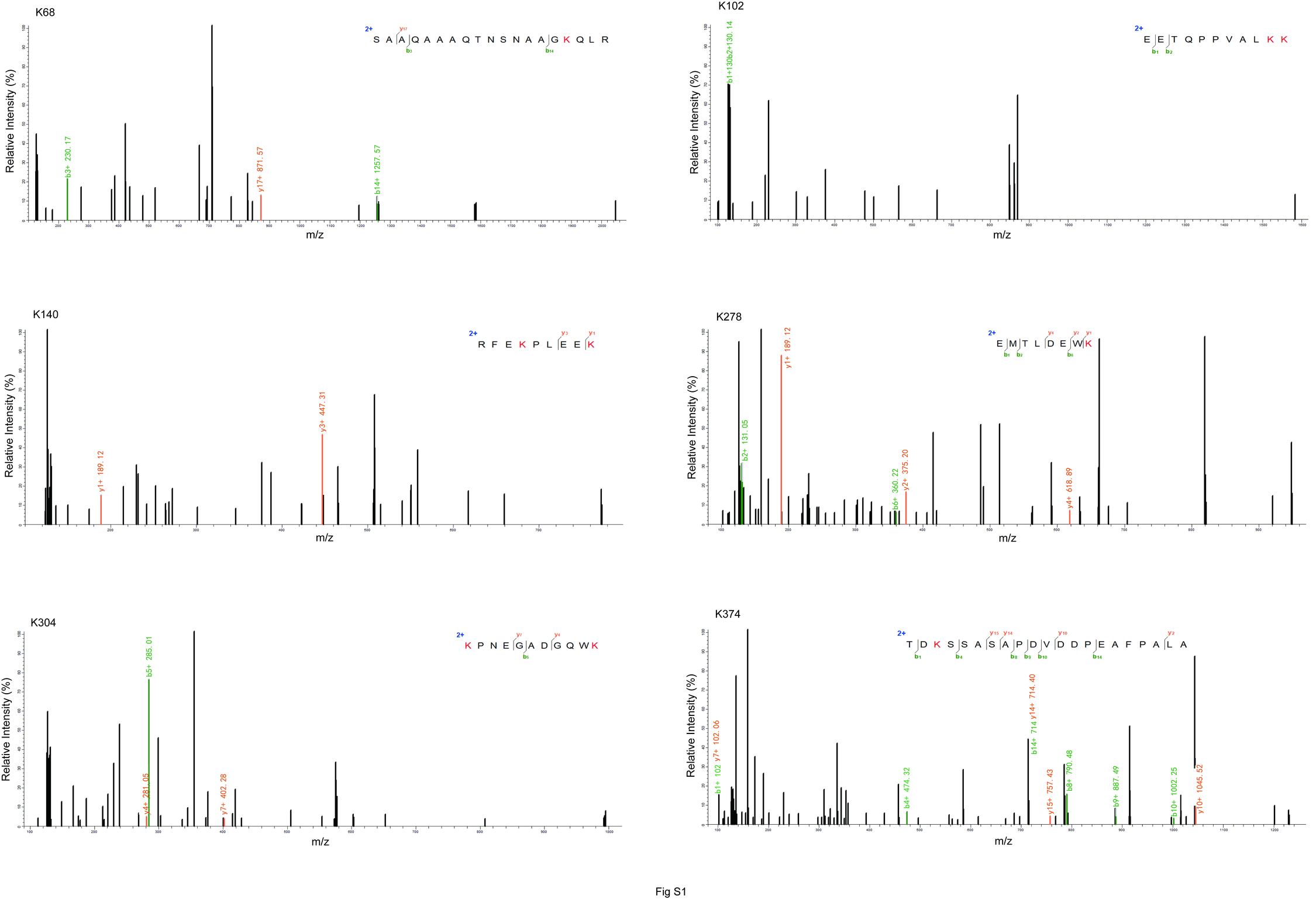

Supplement: S1 Fig — Acetylation of SERBP1 lysine residues at Lys68, 102, 140, 278, 304, 374 were identified in both MM and KMM cells by tandem mass tag (TMT) labeling proteomic technique. The b and y ions in the spectra of the peptide were marked in green and orange, respectively. (TIF) [file ppat.1012082.s001.tif]

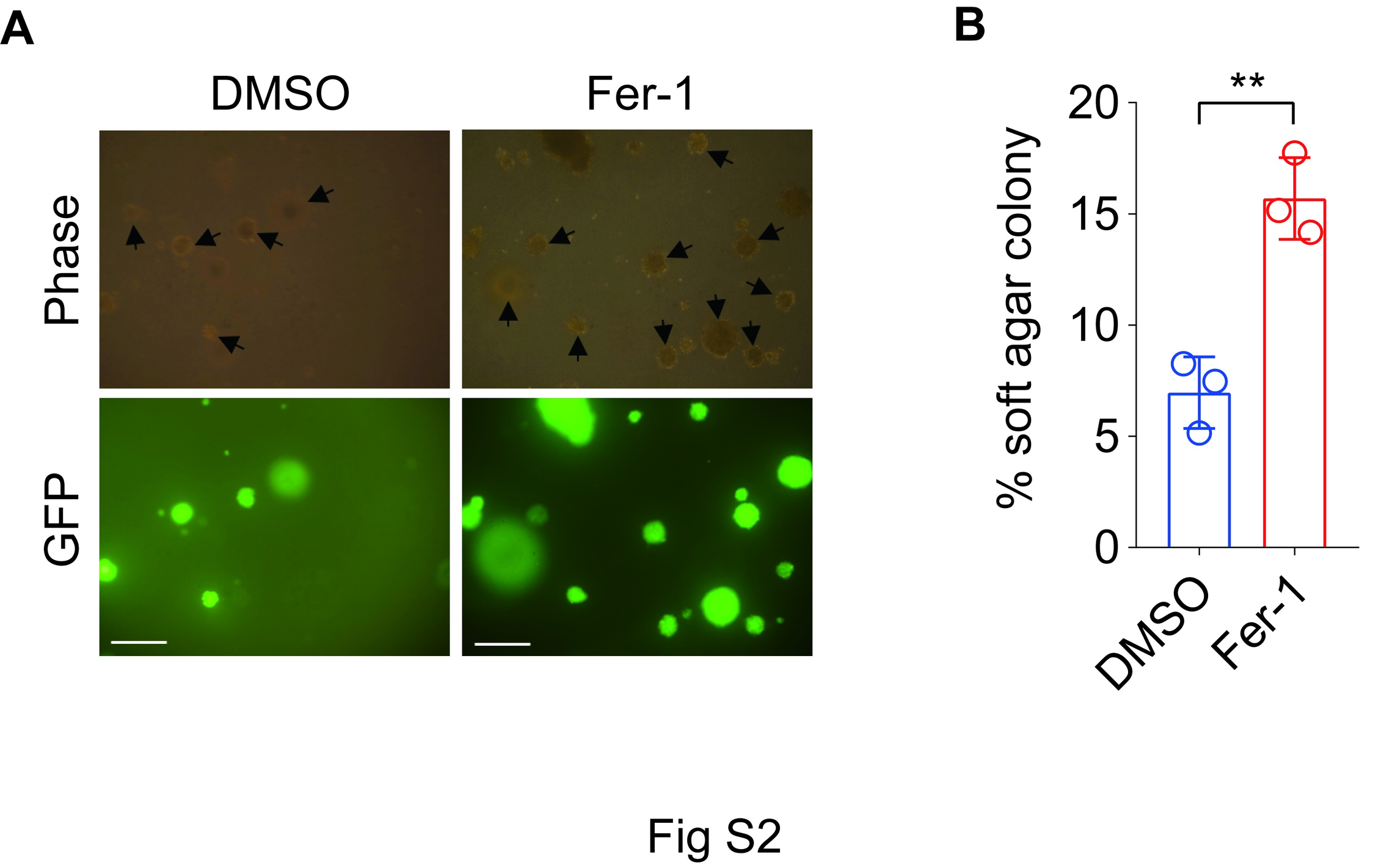

Supplement: S2 Fig — (A). Soft agar assay of KMM cells treated with 10 μM Fer-1 for 24h. The representative images were captured at 2 weeks post seeding. Magnification, ×100. Scar bars, 40 μm. The representative images were taken from three randomly selected fields of each sample. (B). Quantification of the results in (A). Colonies with a size equal to or larger than 20 μm (arrows shown in A) were counted to calculate the percentage of soft agar colonies. (TIF) [file ppat.1012082.s002.tif]

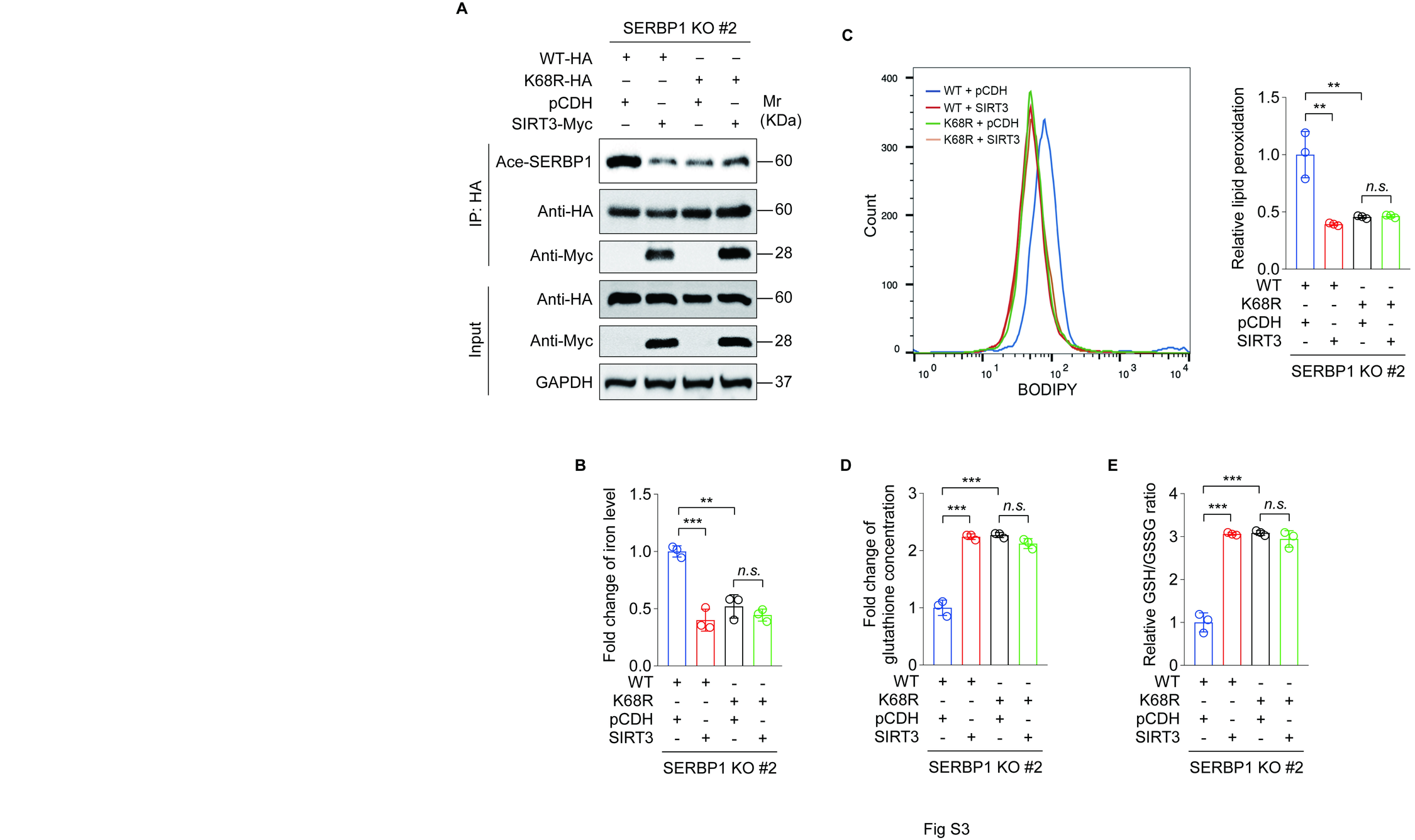

Supplement: S3 Fig — (A). SERBP1 KO KMM cells infected with lentiviral SERBP1 WT-HA (WT-HA) or SERBP1 K68R-HA (K68R-HA) were transduced with lentiviral SIRT3-Myc (SIRT3-Myc) or its control pCDH (pCDH). The acetylation of SERBP1 was examined by immunoprecipitating with anti-HA antibody. (B). Levels of iron in cells treated as in (A). (C). Flow cytometry analysis of lipid peroxidation level in cells treated as in (A) (left). The lipid peroxidation level of indicated cells was shown in bar graph (right). (D). Levels of total glutathione in cells treated as in (A). (E). Levels of GSH/GSSG ratio in cells treated as in (A). Data were shown as mean ± SD. **P < 0.01 and ***P < 0.001, Student’s t-test. n.s., not significant. (TIF) [file ppat.1012082.s003.tif]

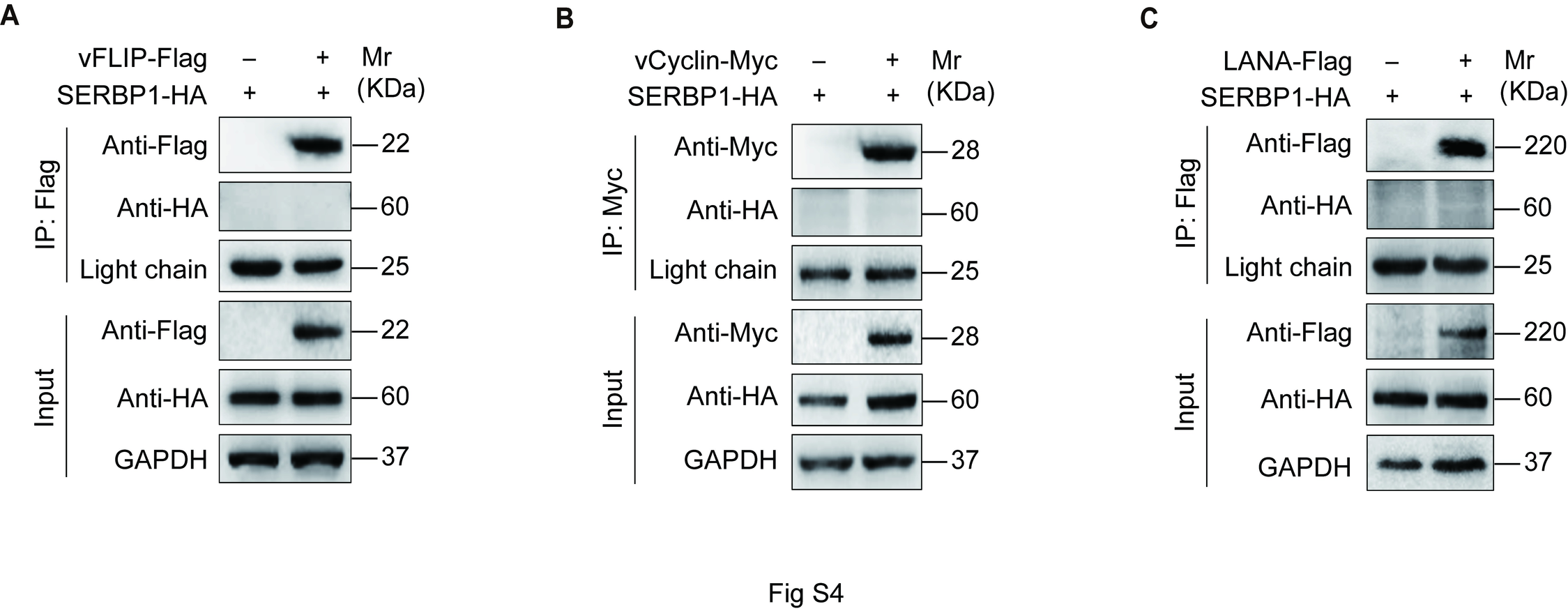

Supplement: S4 Fig — (A). KMM cells infected with lentiviral SERBP1-HA (SERBP1-HA) were transduced with lentiviral vFLIP-Flag (vFLIP-Flag) or its control pHAGE. The interaction between vFLIP and SERBP1 proteins was examined by immunoprecipitating with anti-Flag antibody. (B). KMM cells infected with lentiviral SERBP1-HA (SERBP1-HA) were transduced with lentiviral vCyclin-Myc (vCyclin-Myc) or its control pCDH. The interaction between vCyclin and SERBP1 proteins was examined by immunoprecipitating with anti-Myc antibody. (C). KMM cells infected with lentiviral SERBP1-HA (SERBP1-HA) were transduced with lentiviral LANA-Flag (LANA-Flag) or its control pCDH. The interaction between LANA and SERBP1 proteins was examined by immunoprecipitating with anti-Flag antibody. (TIF) [file ppat.1012082.s004.tif]
