# Supplementary material for: KSHV vIL-6 promotes SIRT3-induced deacetylation of SERBP1 to inhibit ferroptosis and enhance cellular transformation by inducing lipoyltransferase 2 mRNA degradation
Source: PLoS Pathog. 2024 Mar 12;20(3):e1012082. doi: 10.1371/journal.ppat.1012082 (PMC10959363; doi:10.1371/journal.ppat.1012082)
Supplement: S1 Table — (PDF) [file ppat.1012082.s005.pdf]

S1 Table. The sequences of the sgRNAs.

Target	Sequence
SERBP1-sgRNA	CGGCGTGGCTGACAAAAAGG
SIRT3-sgRNA-1	CCAACGTGAAAAAGGGCTTG
SIRT3-sgRNA-2	GTATGACATCCCGTACCCTG
SIRT3-sgRNA-3	CCTTGTCGTGAAGCAGCCGA
