# Supplementary material for: KSHV vIL-6 promotes SIRT3-induced deacetylation of SERBP1 to inhibit ferroptosis and enhance cellular transformation by inducing lipoyltransferase 2 mRNA degradation
Source: PLoS Pathog. 2024 Mar 12;20(3):e1012082. doi: 10.1371/journal.ppat.1012082 (PMC10959363; doi:10.1371/journal.ppat.1012082)
Supplement: S2 Table — (PDF) [file ppat.1012082.s006.pdf]

S2 Table. The primers used in this study.

Target	Primer
Lipt2	F: 5′- TGCCACCCGGTGCTTGACCTG -3′<br>R: 5′- CTCGCCTAGCCAGACGCCAGT -3′
Kif2	F: 5′- ACCAACTGCGGCAAGACCTACACC-3′<br>R: 5′- GGCTTCTCACCTGTGTGCGTCCTC-3′
Myom2	F: 5′- TCTCACCTGATGCCAACTTCCG -3′<br>R: 5′- CAATAGTAAACCGATCCATGACCA -3′
Ppm1d	F: 5′- CCATCTCCATGTGCCAAGACCA -3′<br>R: 5′- CTCGAAGCATACGCTGCCTCC -3′
Ntn3	F: 5′- CTCCAAGACTGGGTGACTGCCACA -3′<br>R: 5′- GCATCGACCTCCCACCTGAAGCTC -3′
GAPDH	F: 5′- GGGTGTGAACCACGAGAAAT -3′<br>R: 5′- ACTGTGGTCATGAGCCCTTC -3′
