# Supplementary material for: KSHV vIL-6 promotes SIRT3-induced deacetylation of SERBP1 to inhibit ferroptosis and enhance cellular transformation by inducing lipoyltransferase 2 mRNA degradation
Source: PLoS Pathog. 2024 Mar 12;20(3):e1012082. doi: 10.1371/journal.ppat.1012082 (PMC10959363; doi:10.1371/journal.ppat.1012082)

Figure 1D

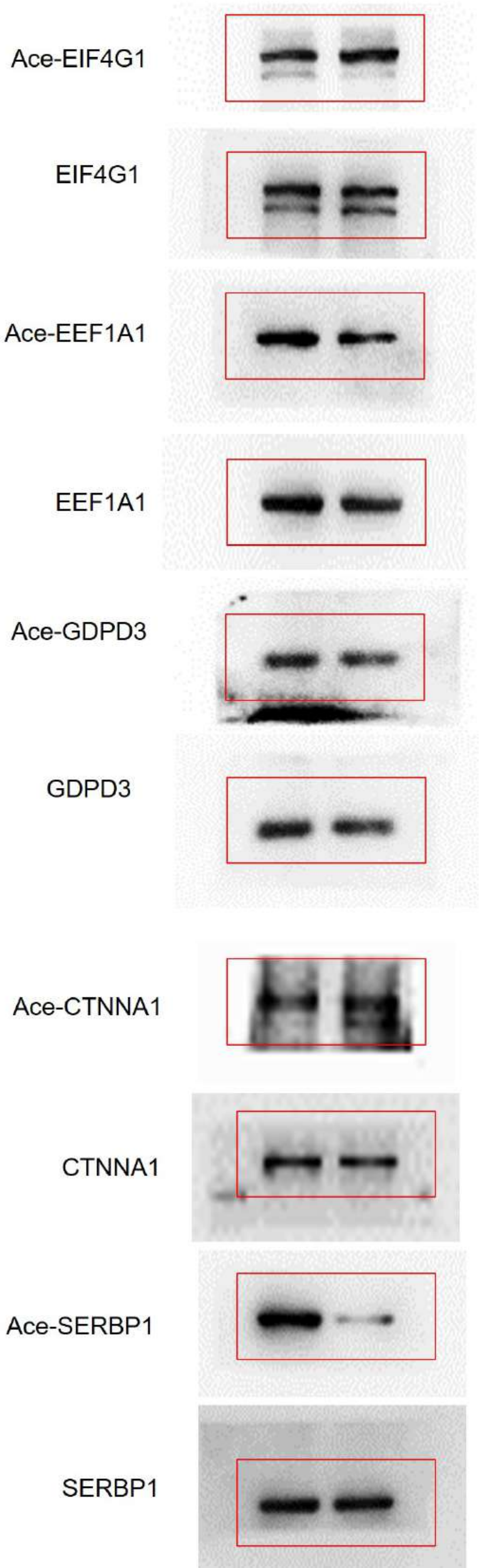

Ace-EIF4G1
EIF4G1
Ace-EEF1A1
EEF1A1
Ace-GDPD3
GDPD3
Ace-CTNNA1
CTNNA1
Ace-SERBP1
SERBP1

Figure 1E

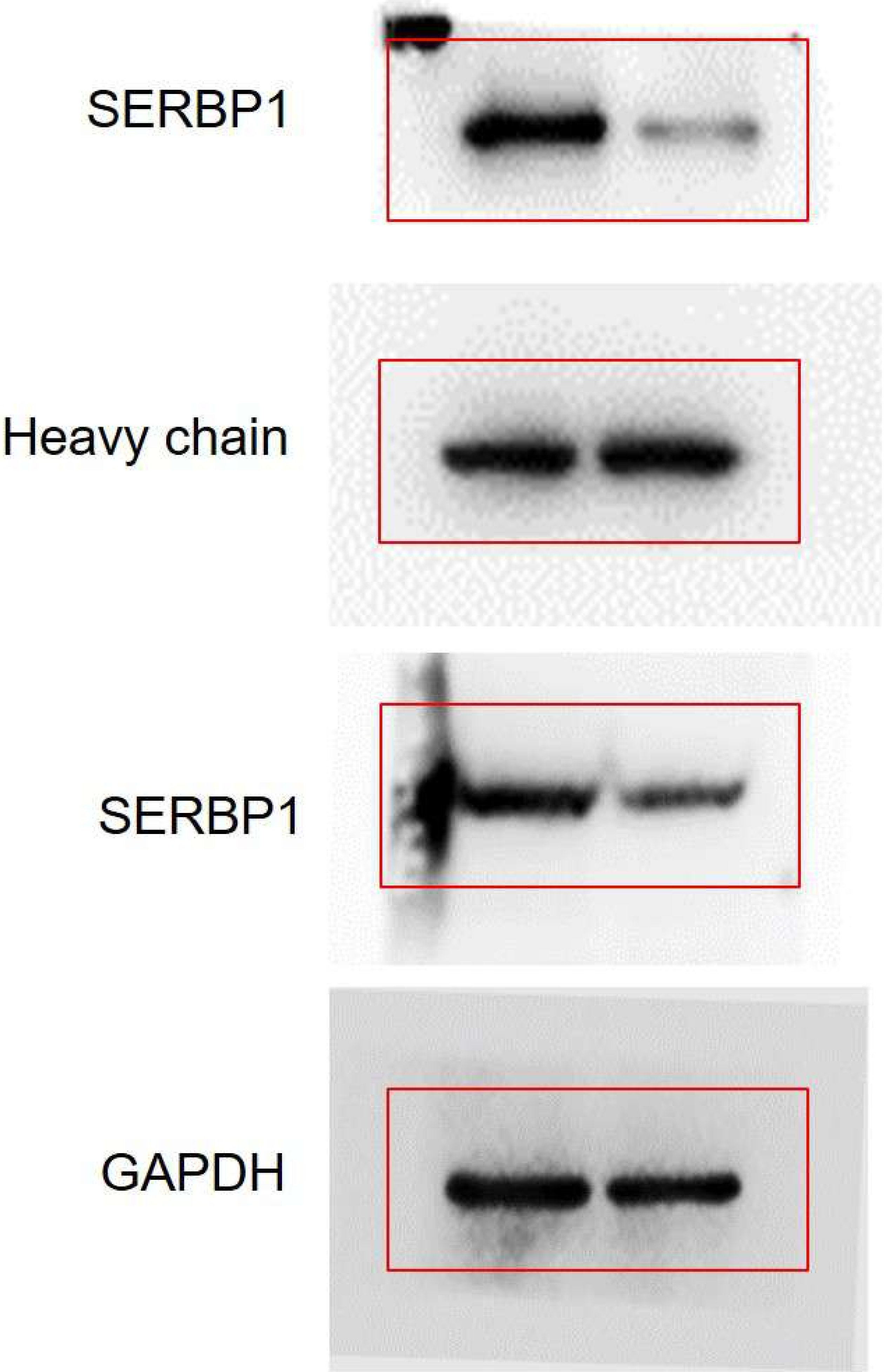

Figure 1F

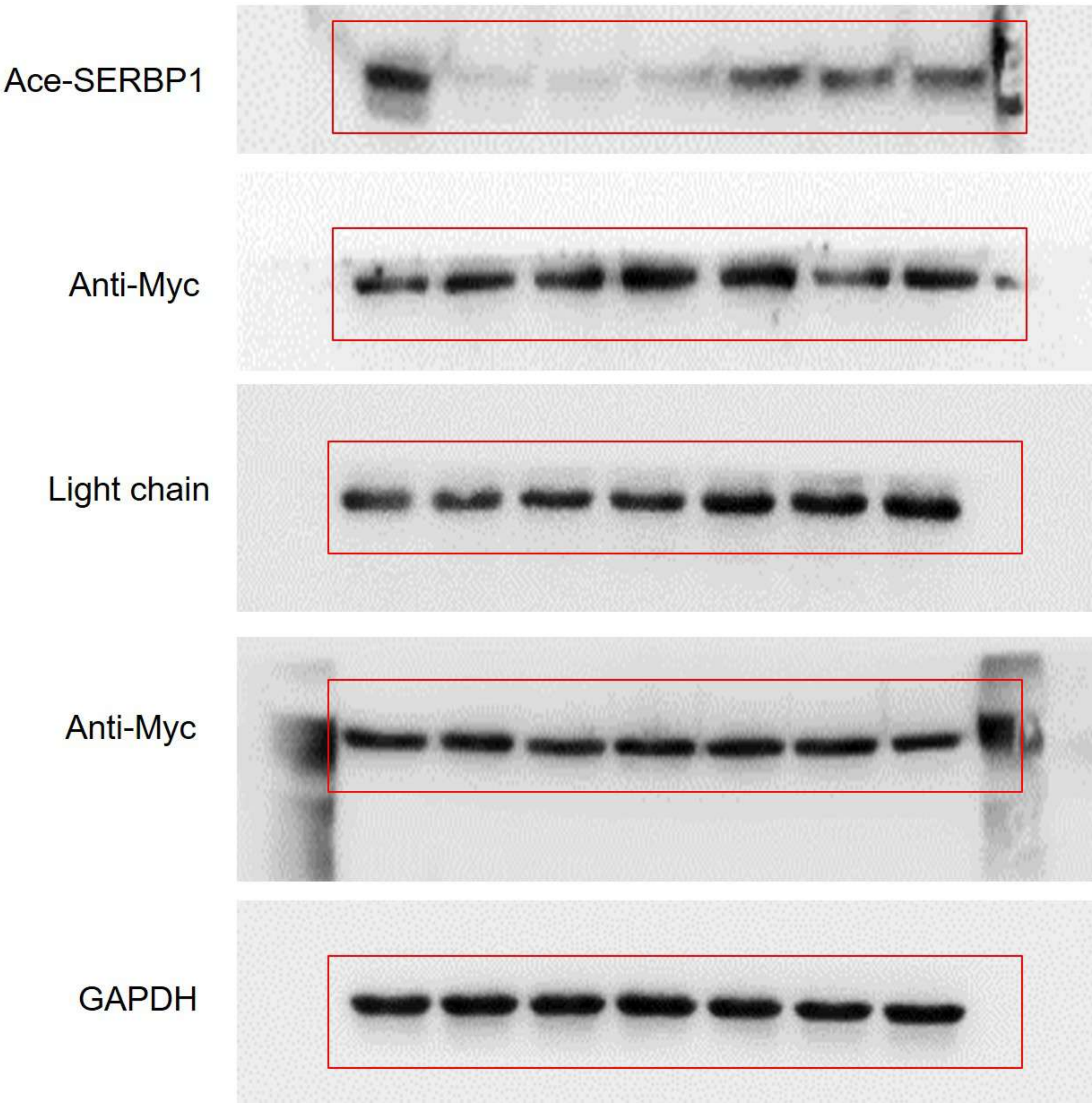

Figure 1G

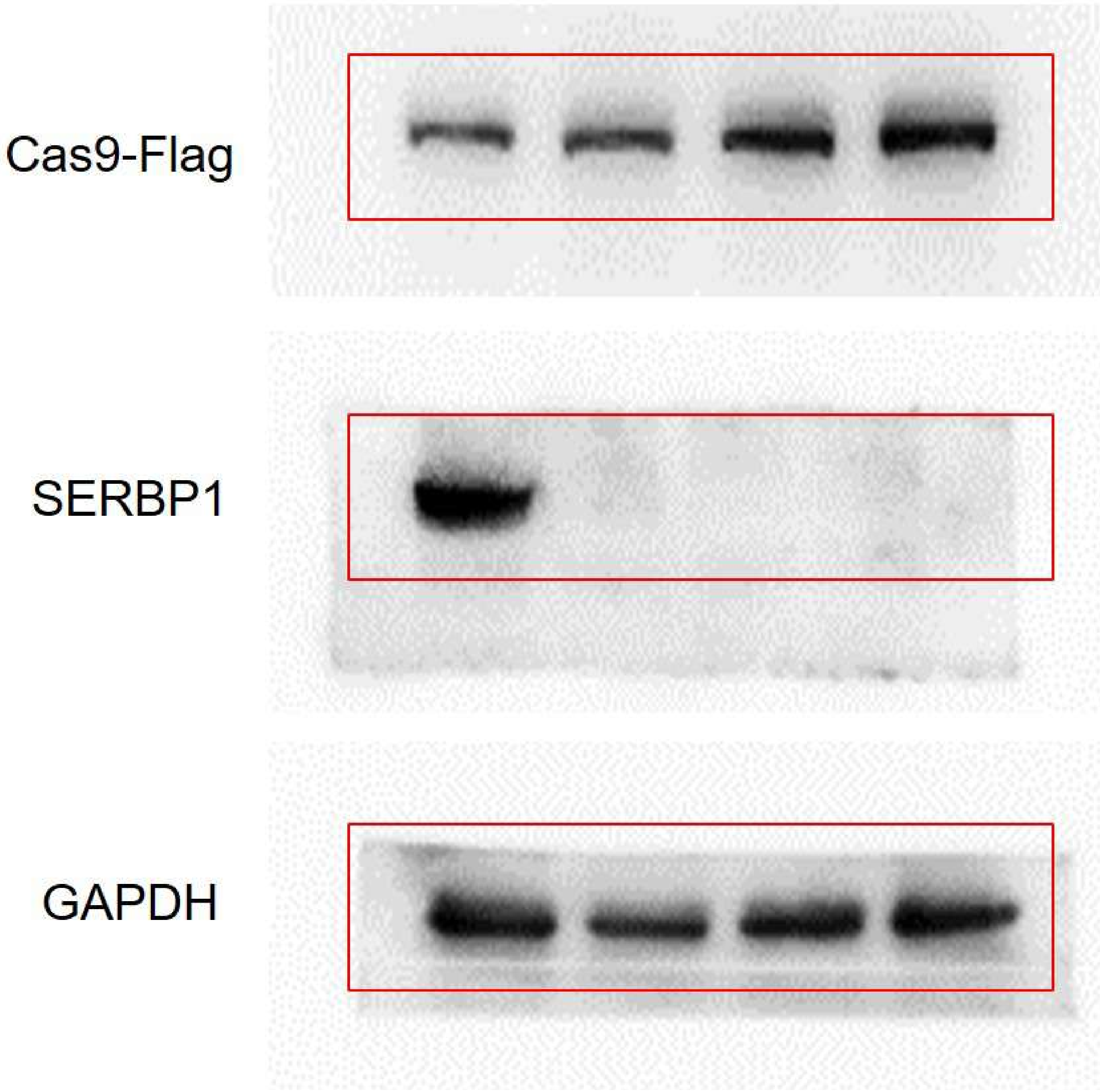

Figure 1L

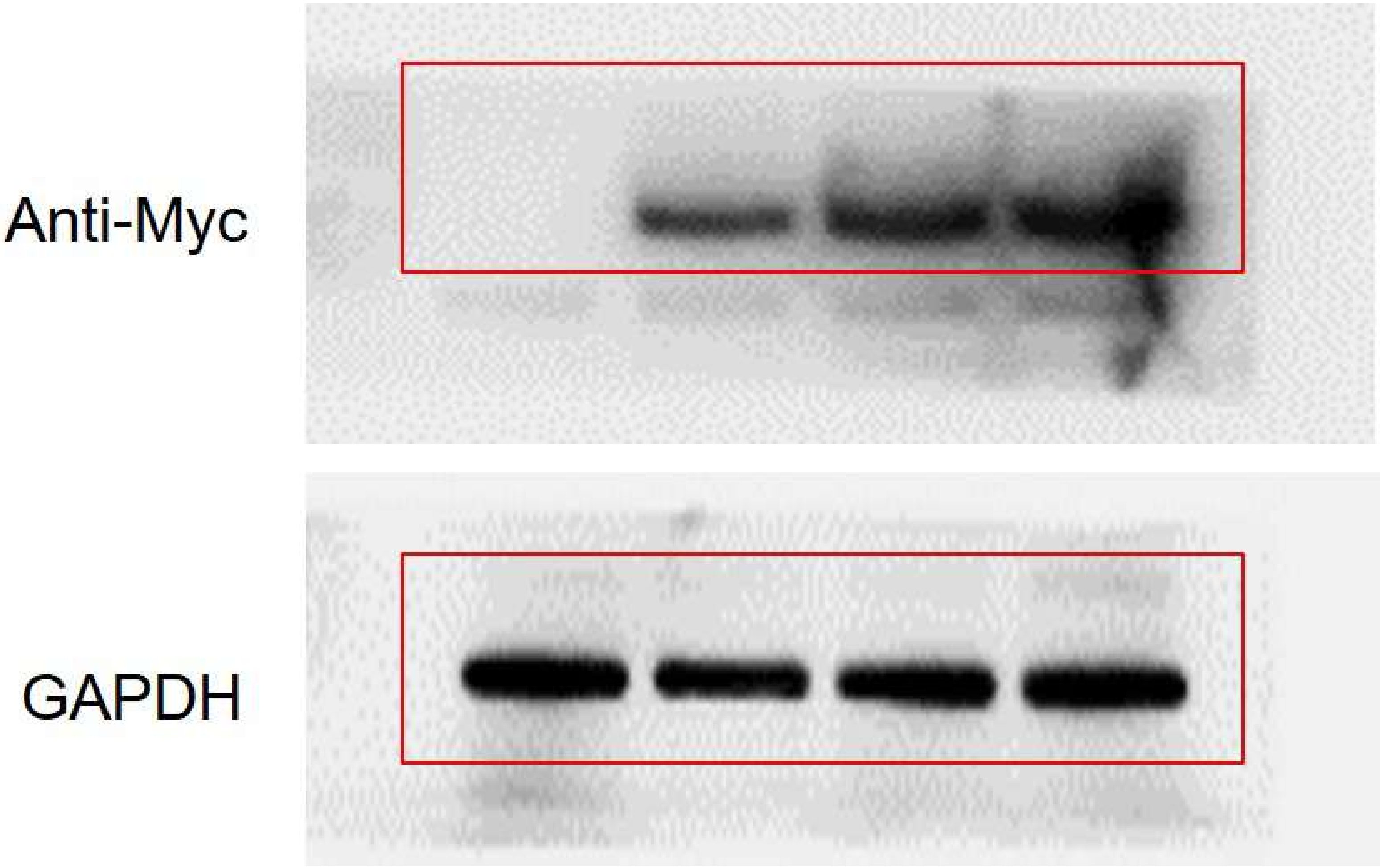

Figure 2E

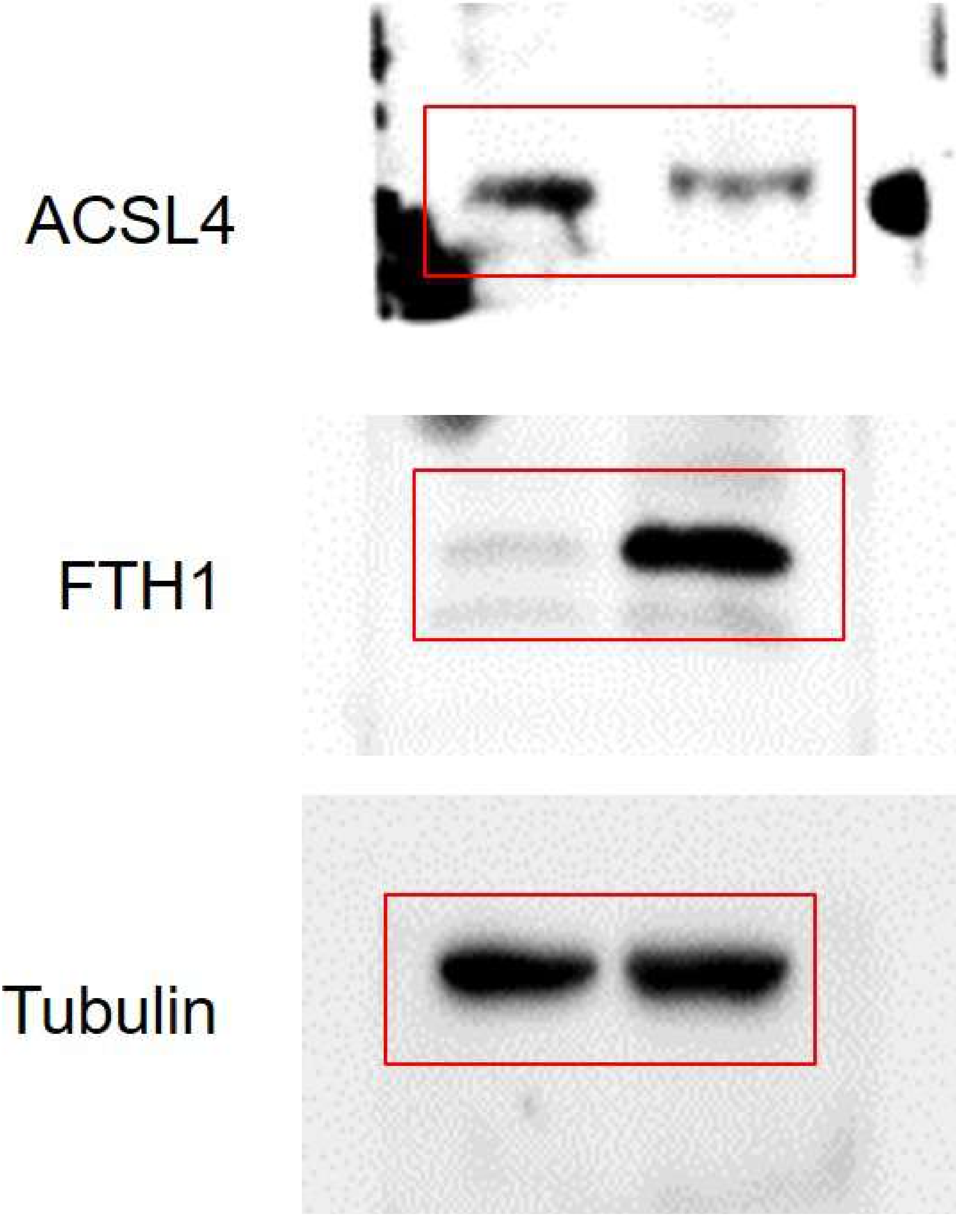

Figure 2J

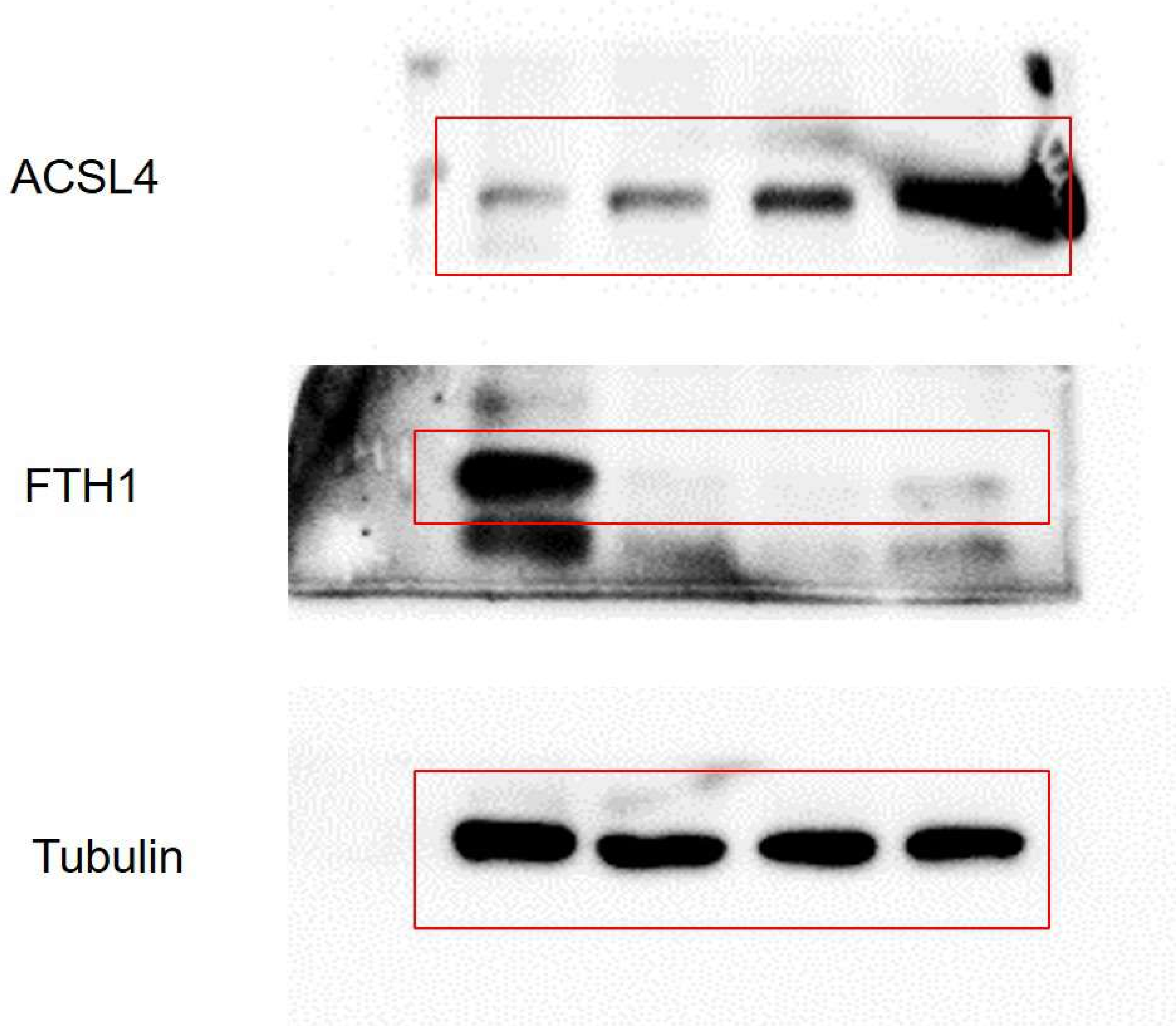

Figure 3A

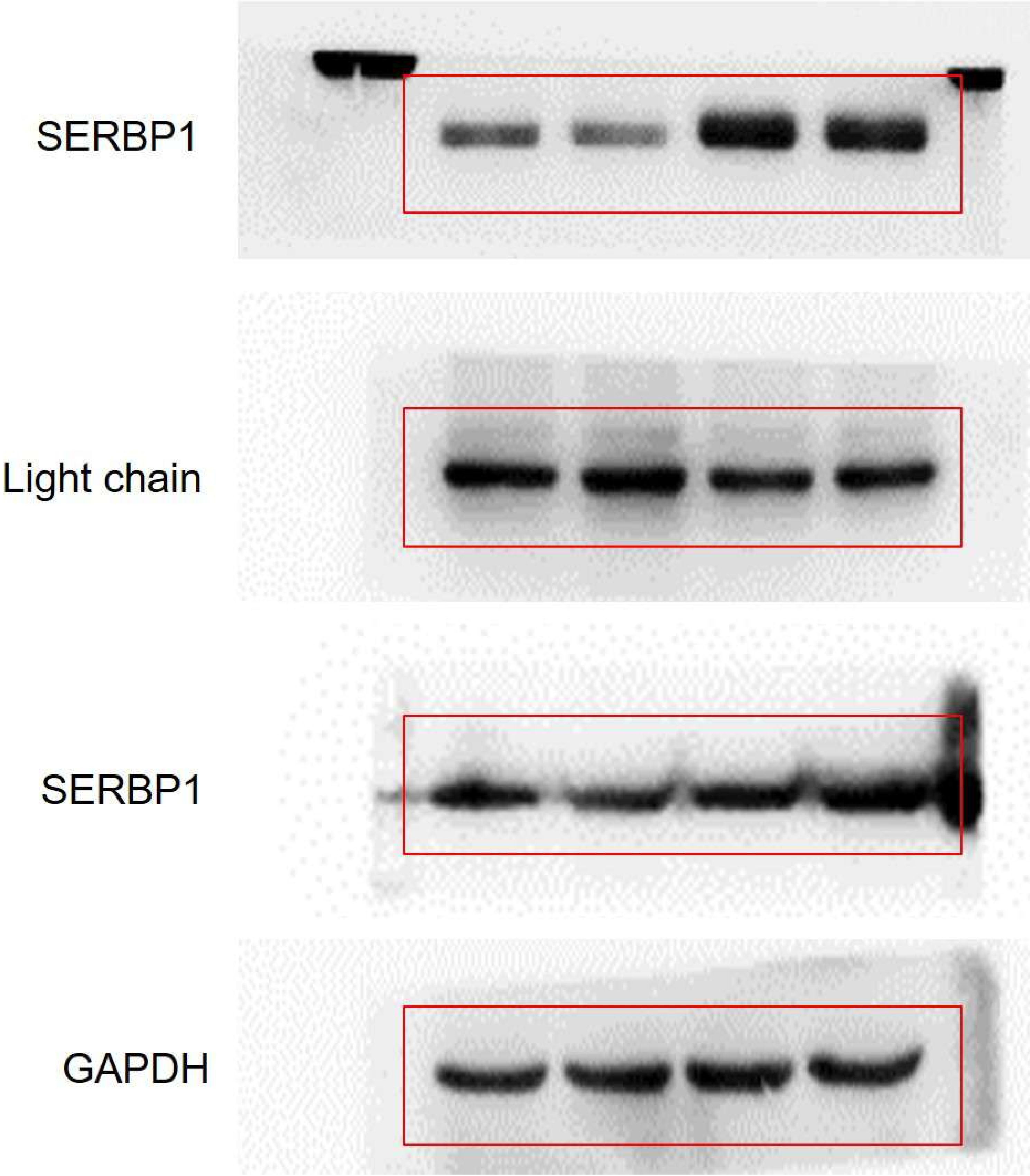

Figure 3B

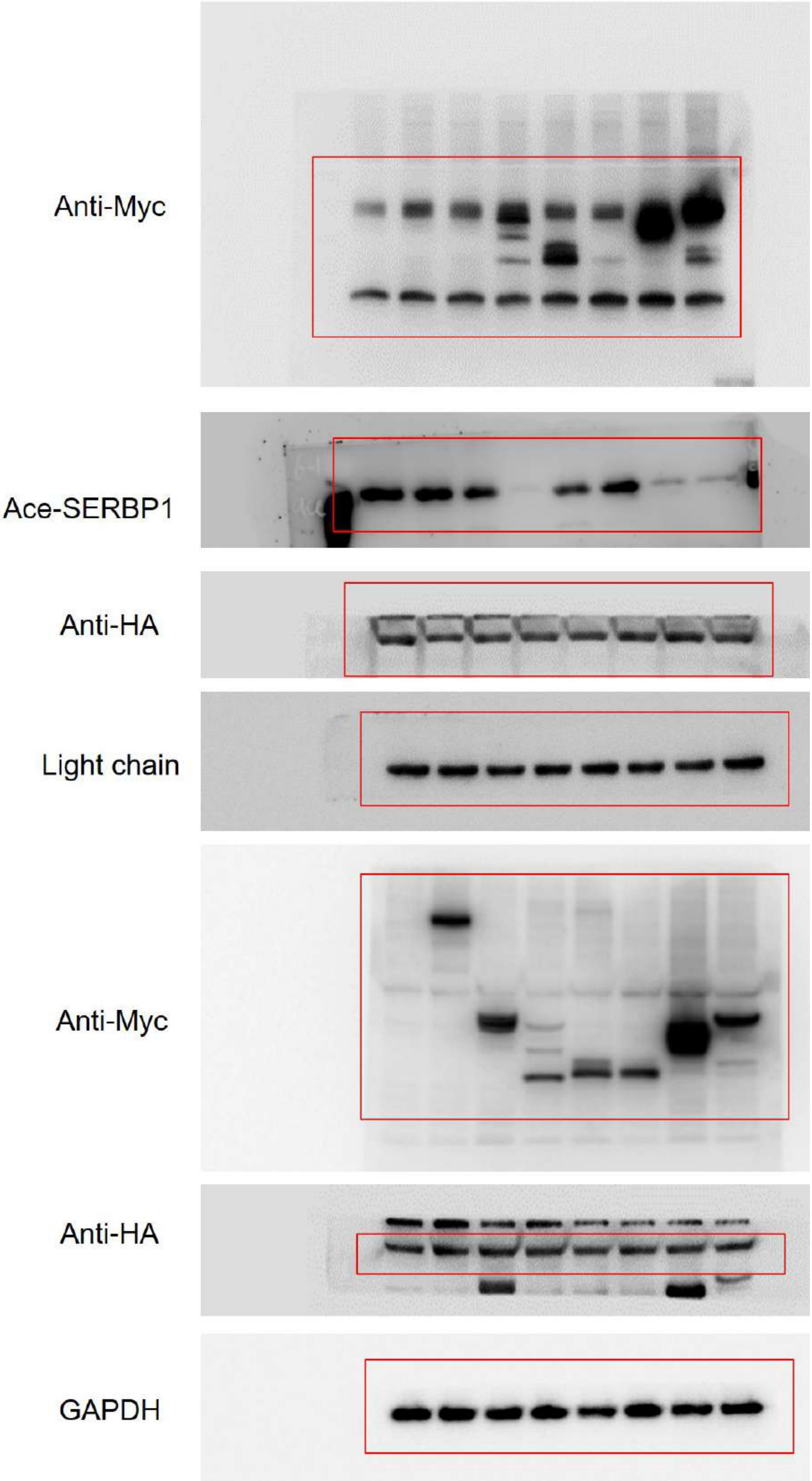
Anti-Myc
Ace-SERBP1
Anti-HA
Light chain
Anti-Myc
Anti-HA
GAPDH

Figure 3C

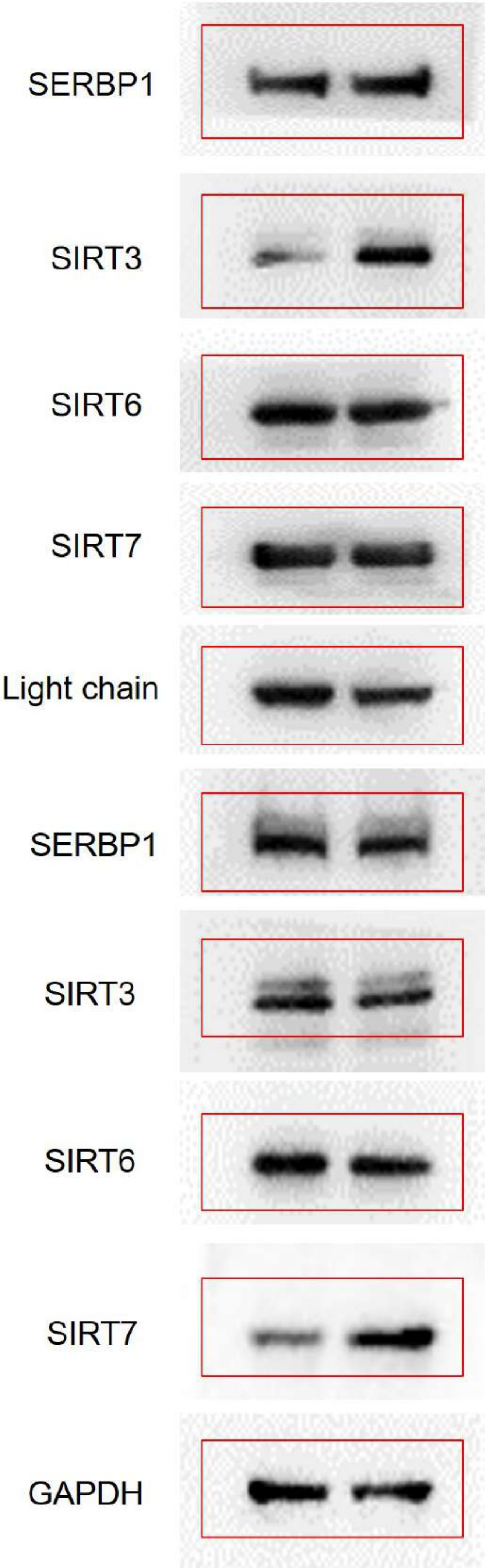
SERBP1
SIRT3
SIRT6
SIRT7
Light chain
SERBP1
SIRT3
SIRT6
SIRT7
GAPDH

Figure 3D

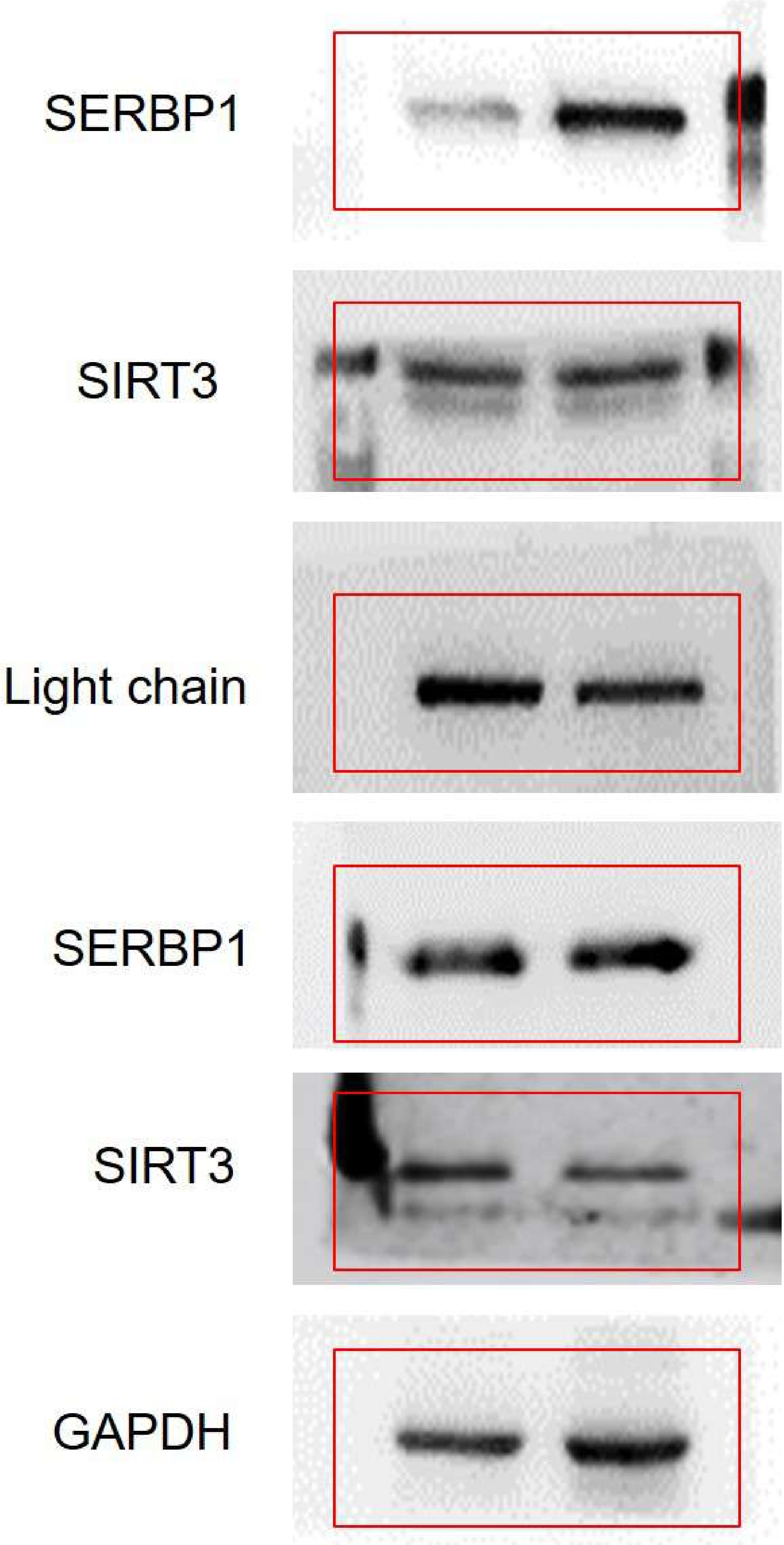

Figure 3E

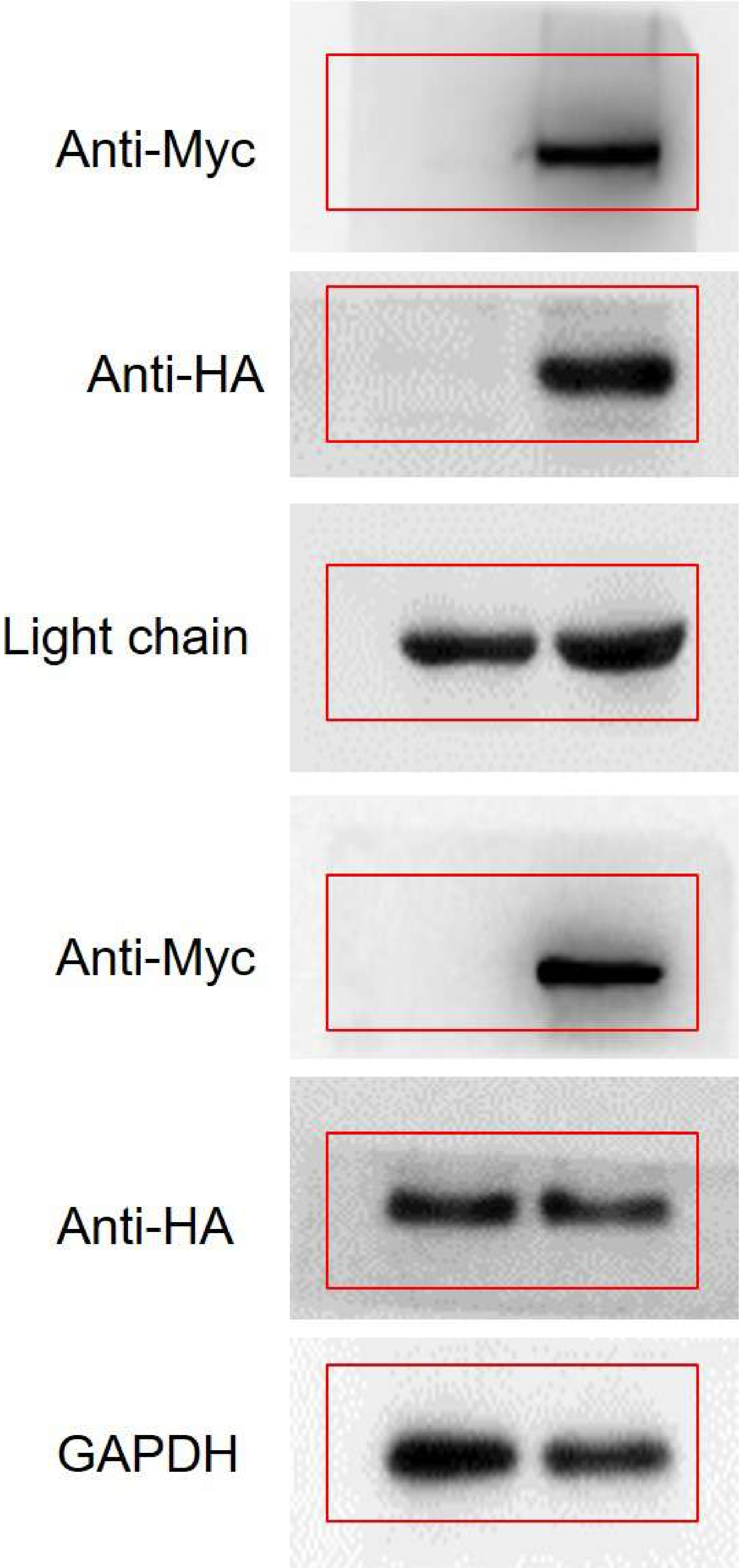

Figure 3F

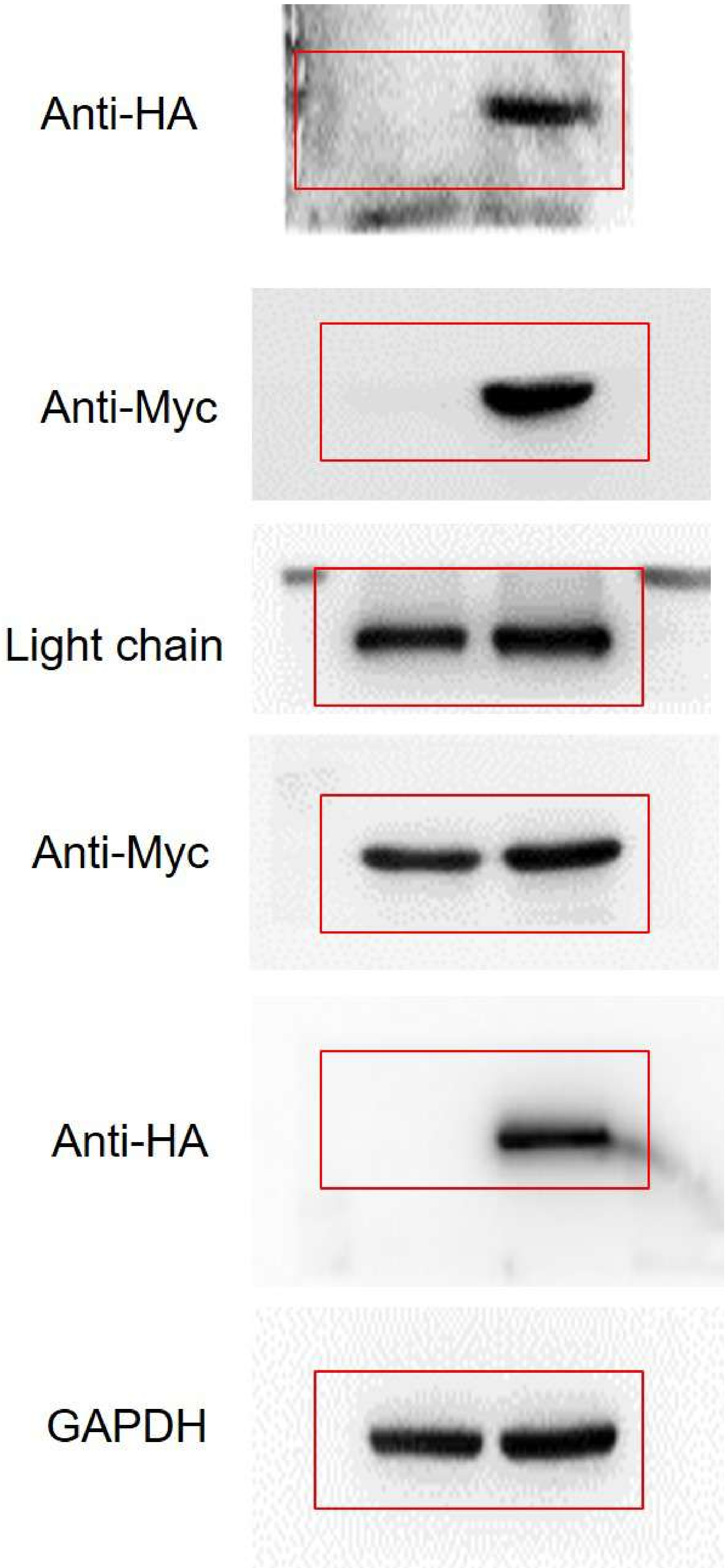

Figure 4A

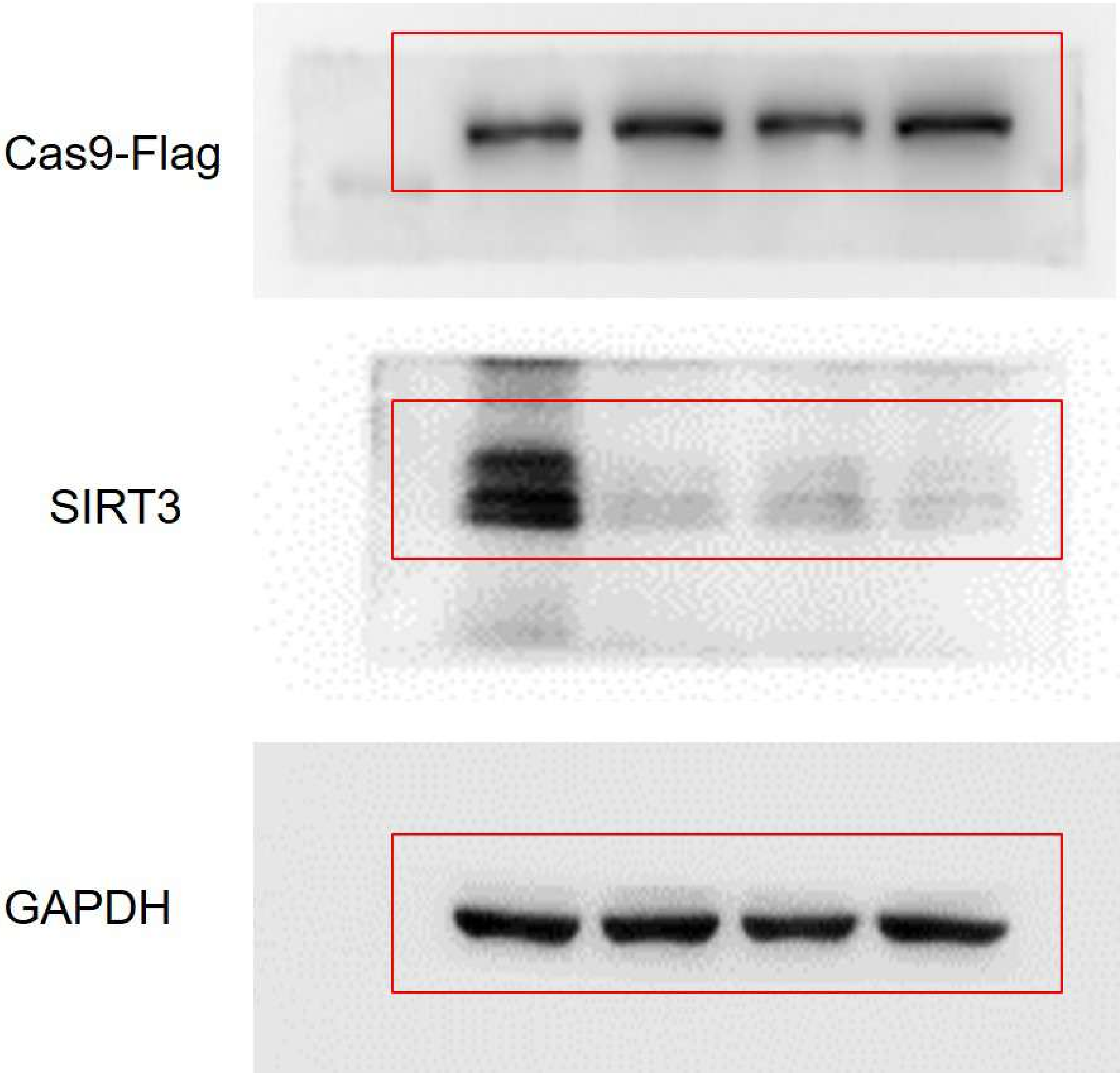

Figure 4B

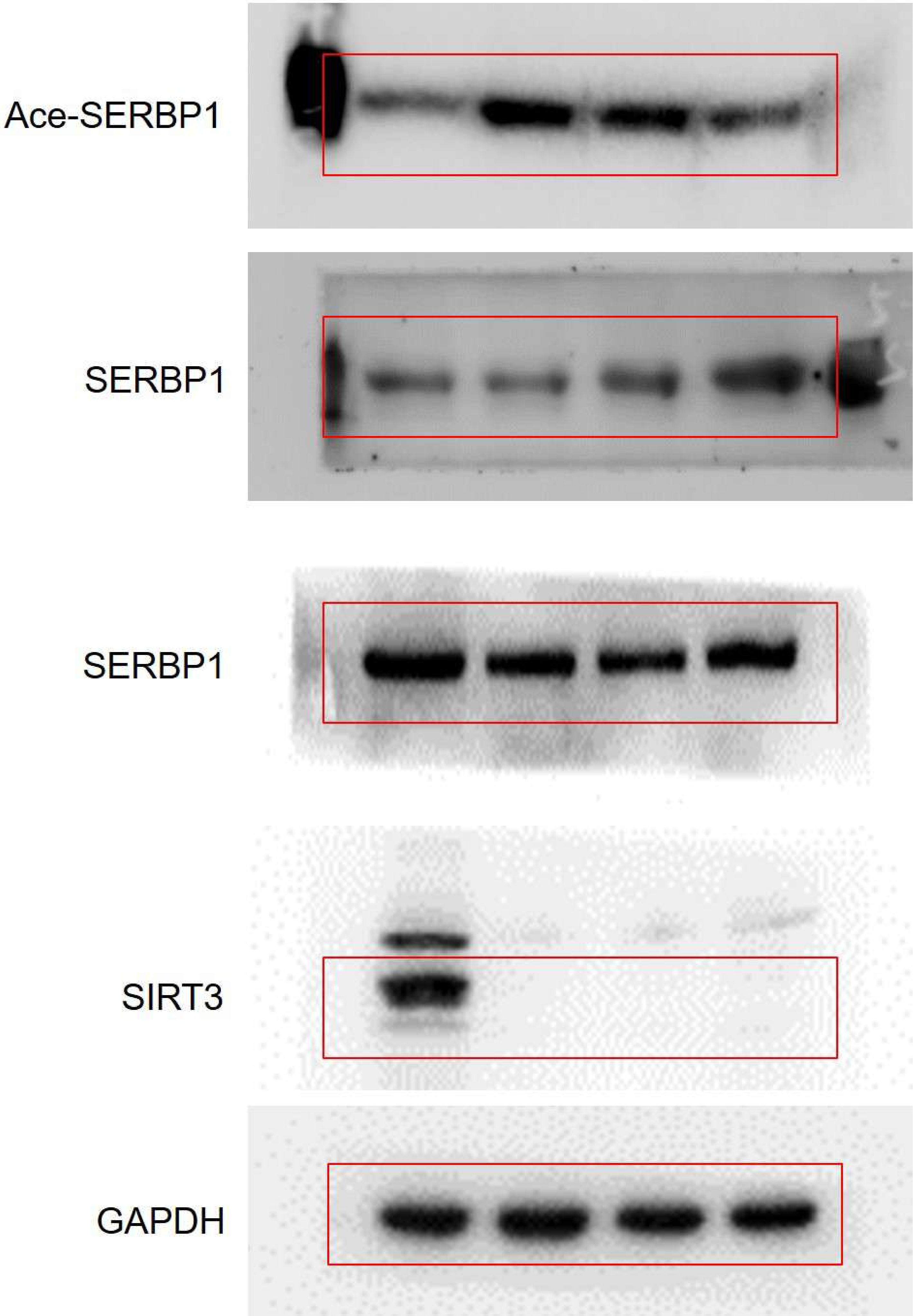

Figure 4G

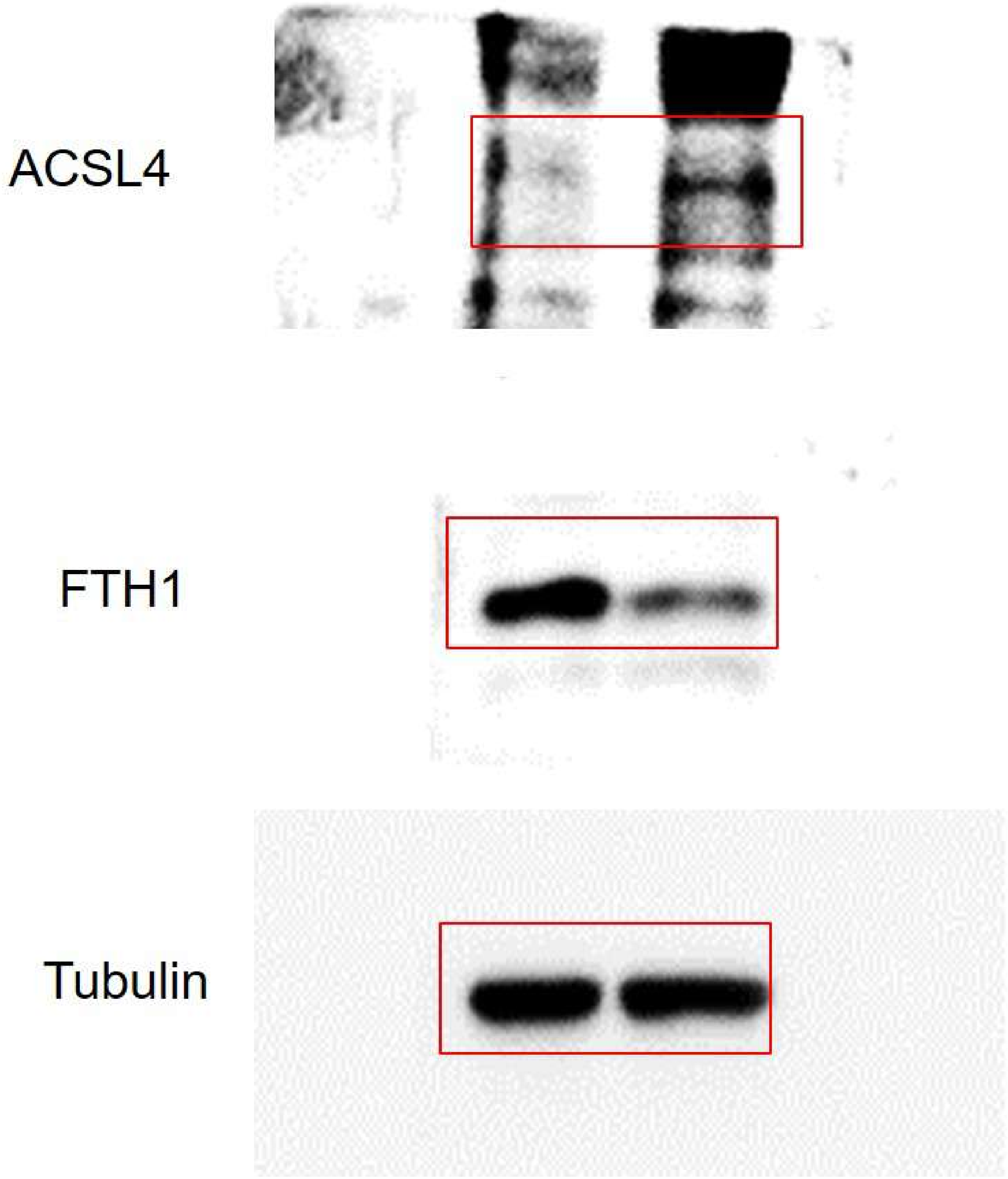

Figure 4J

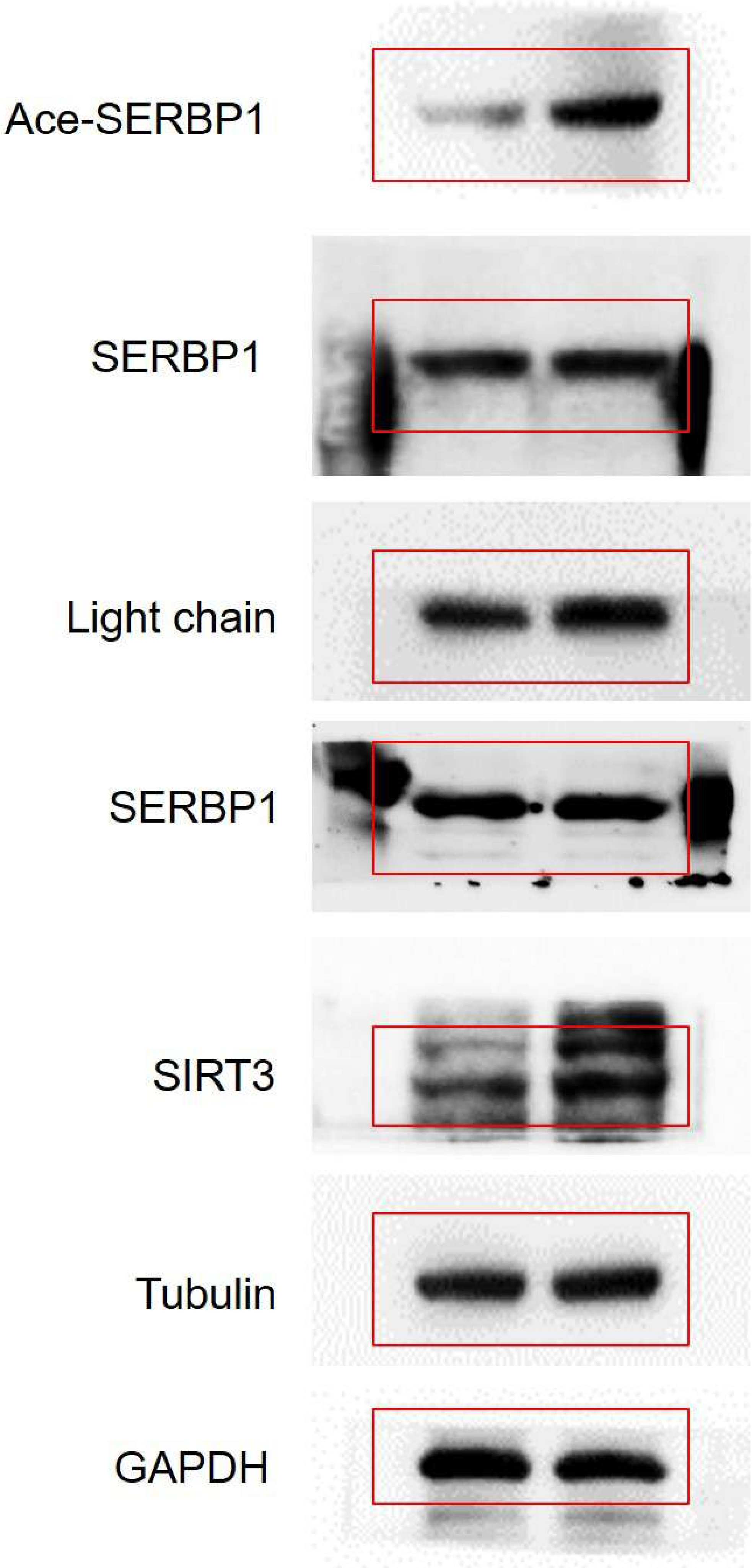

Figure 4K

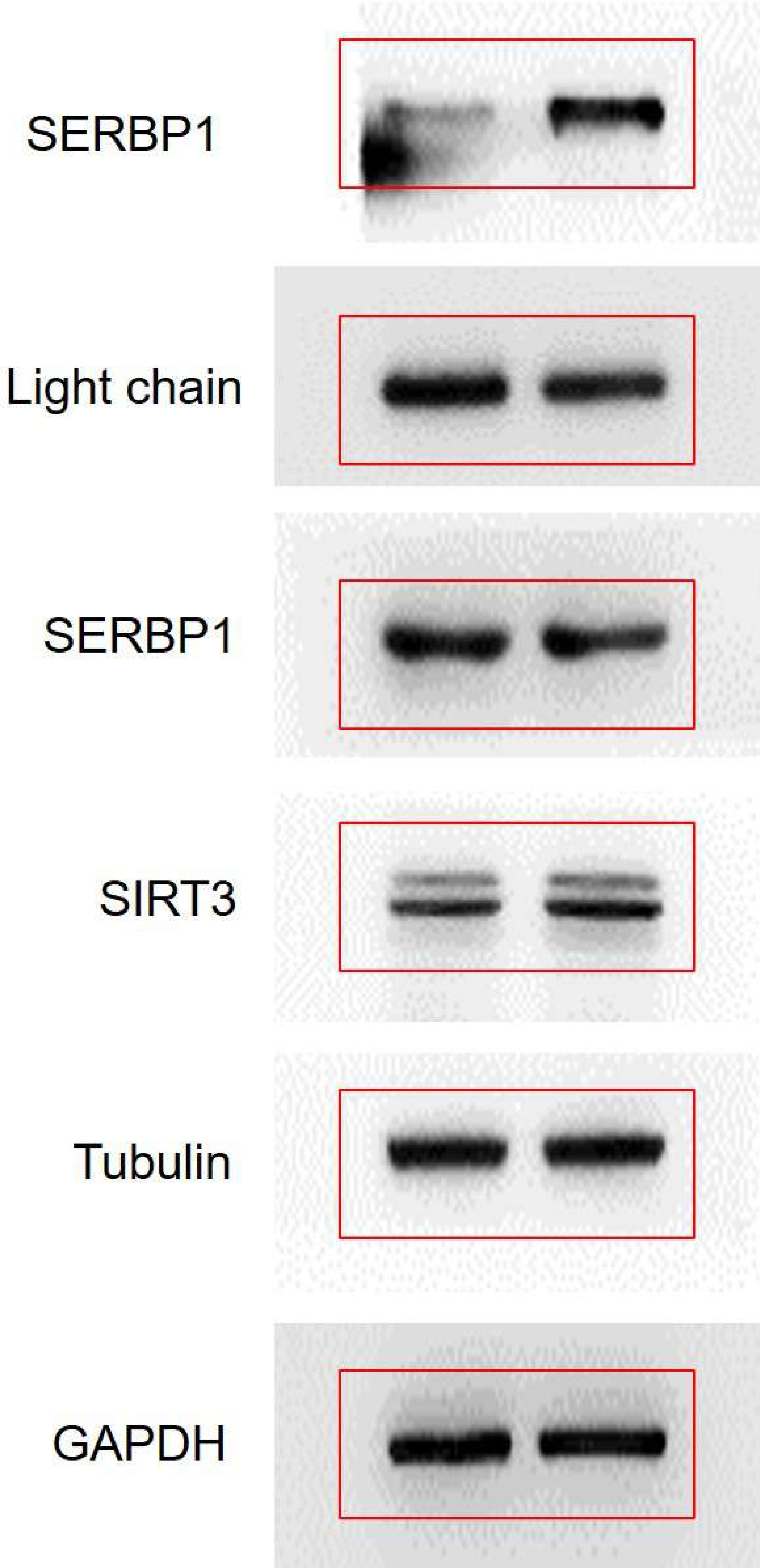

Figure 5A

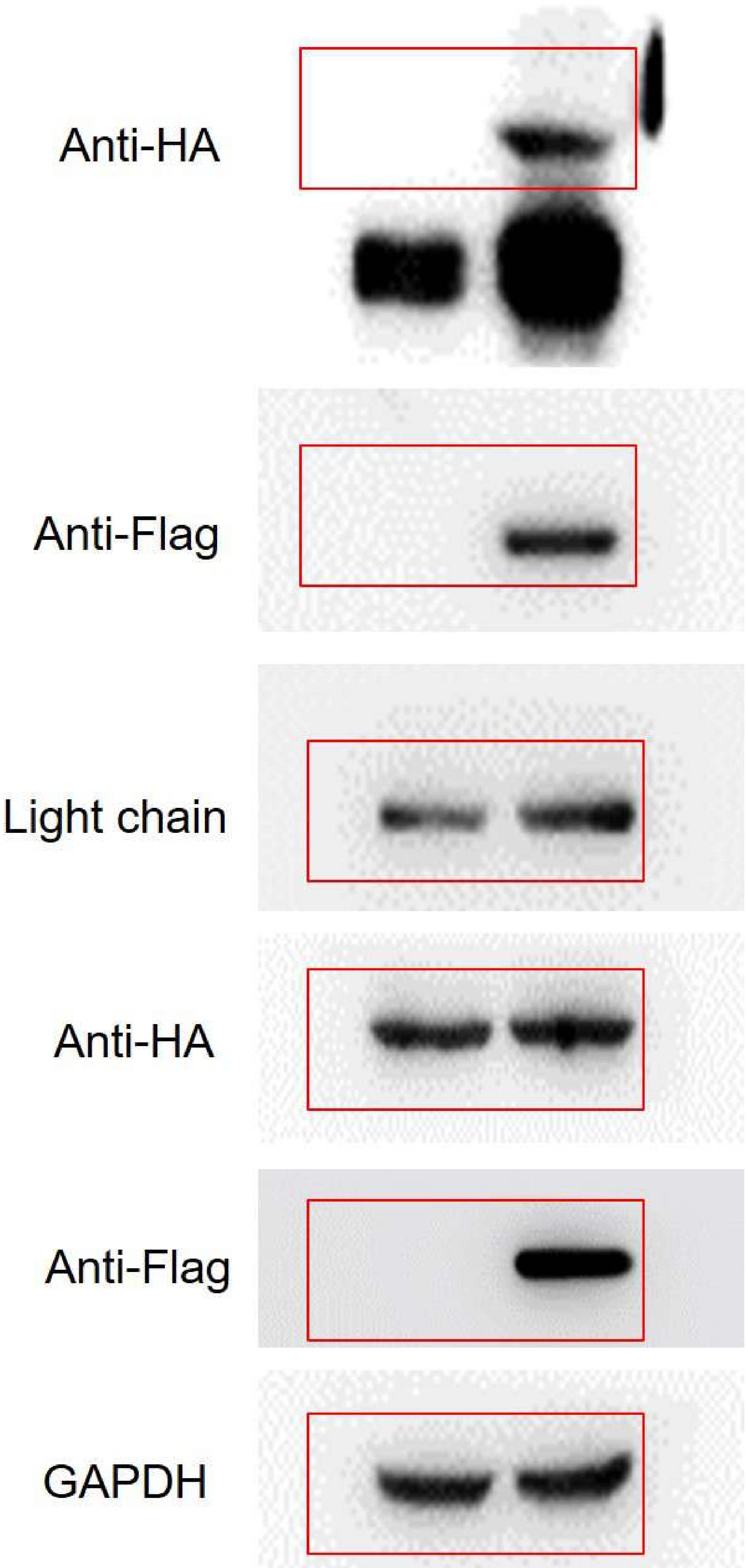

Figure 5B

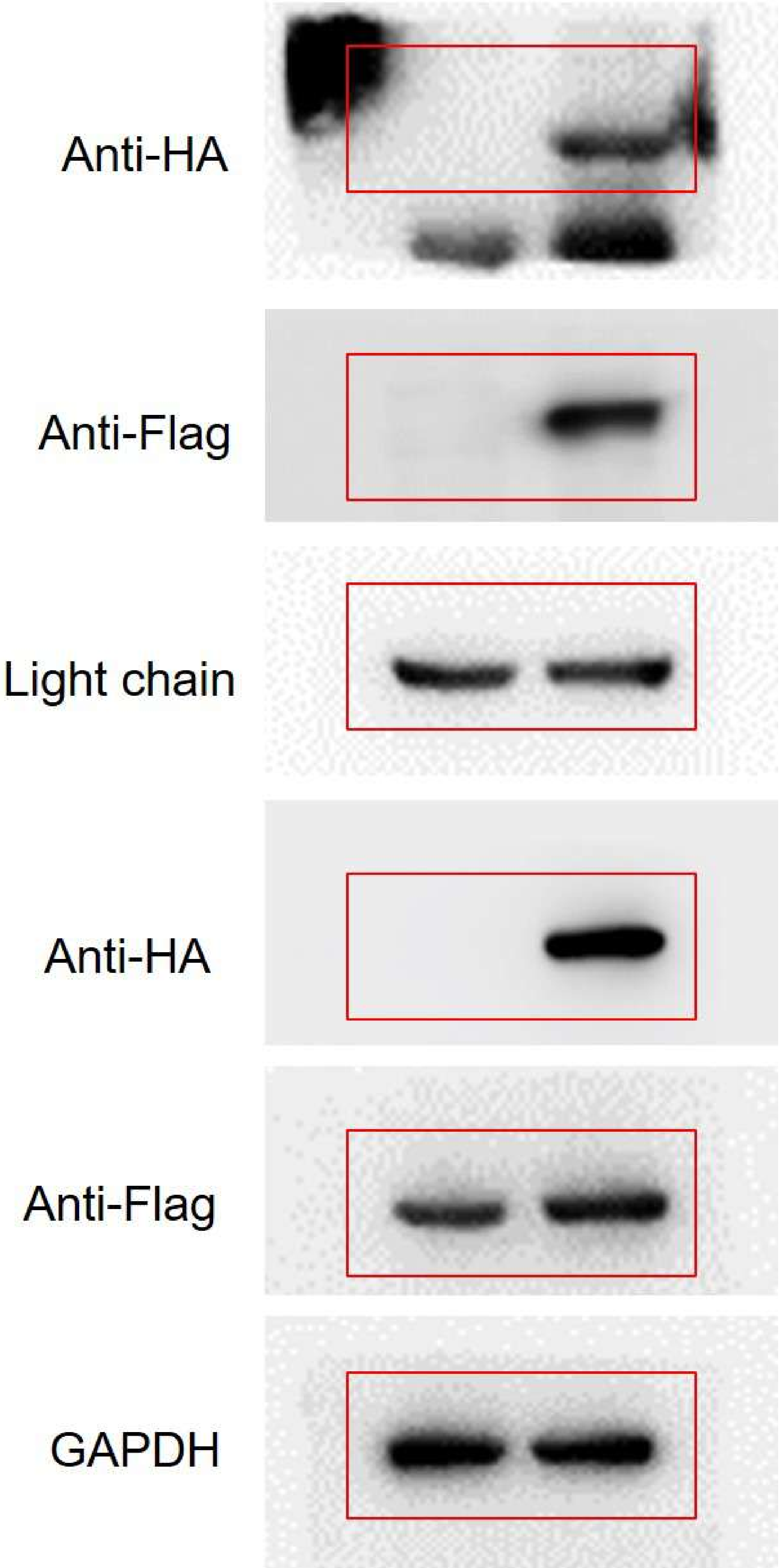

Figure 5C

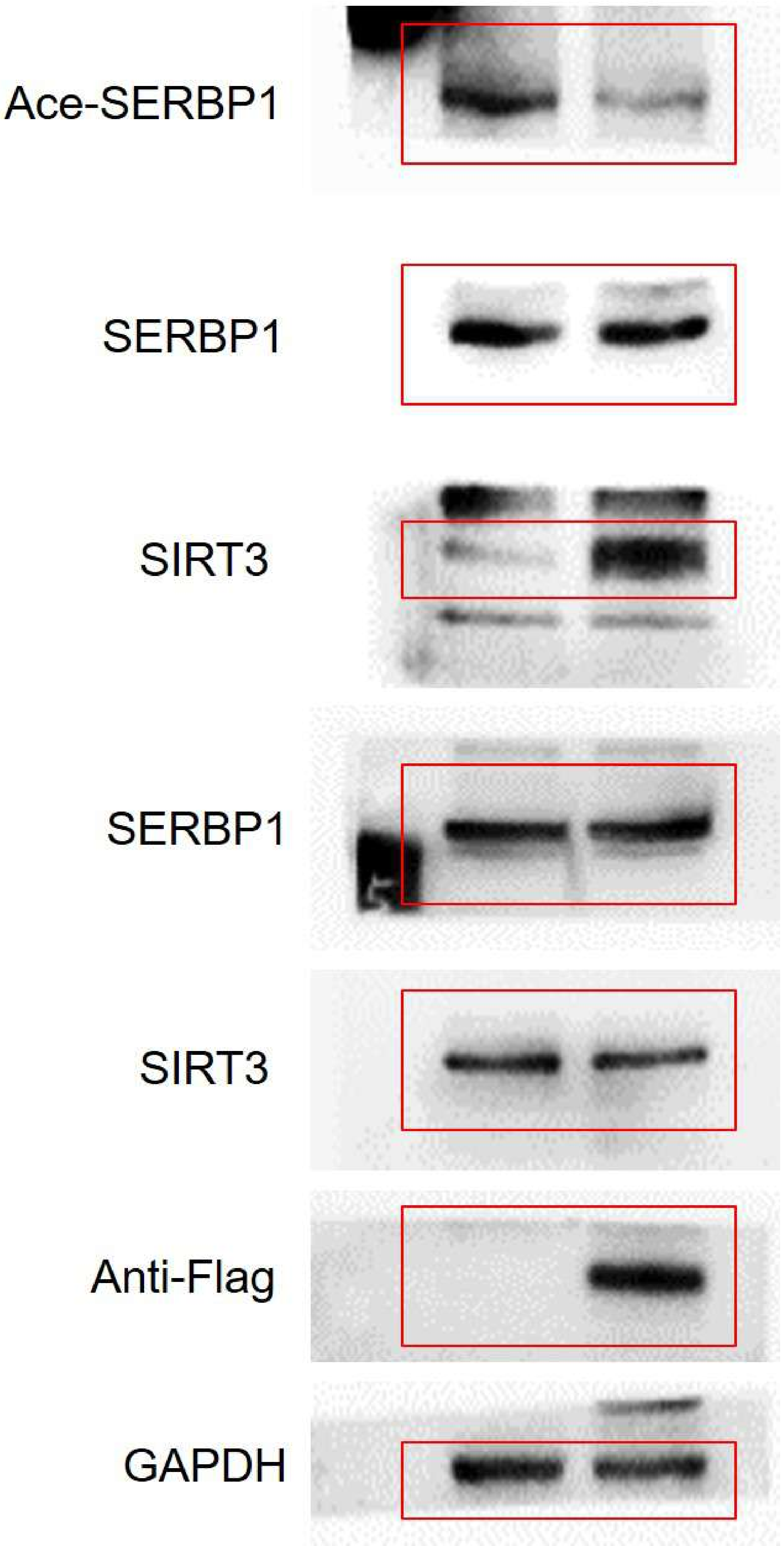

Figure 5D

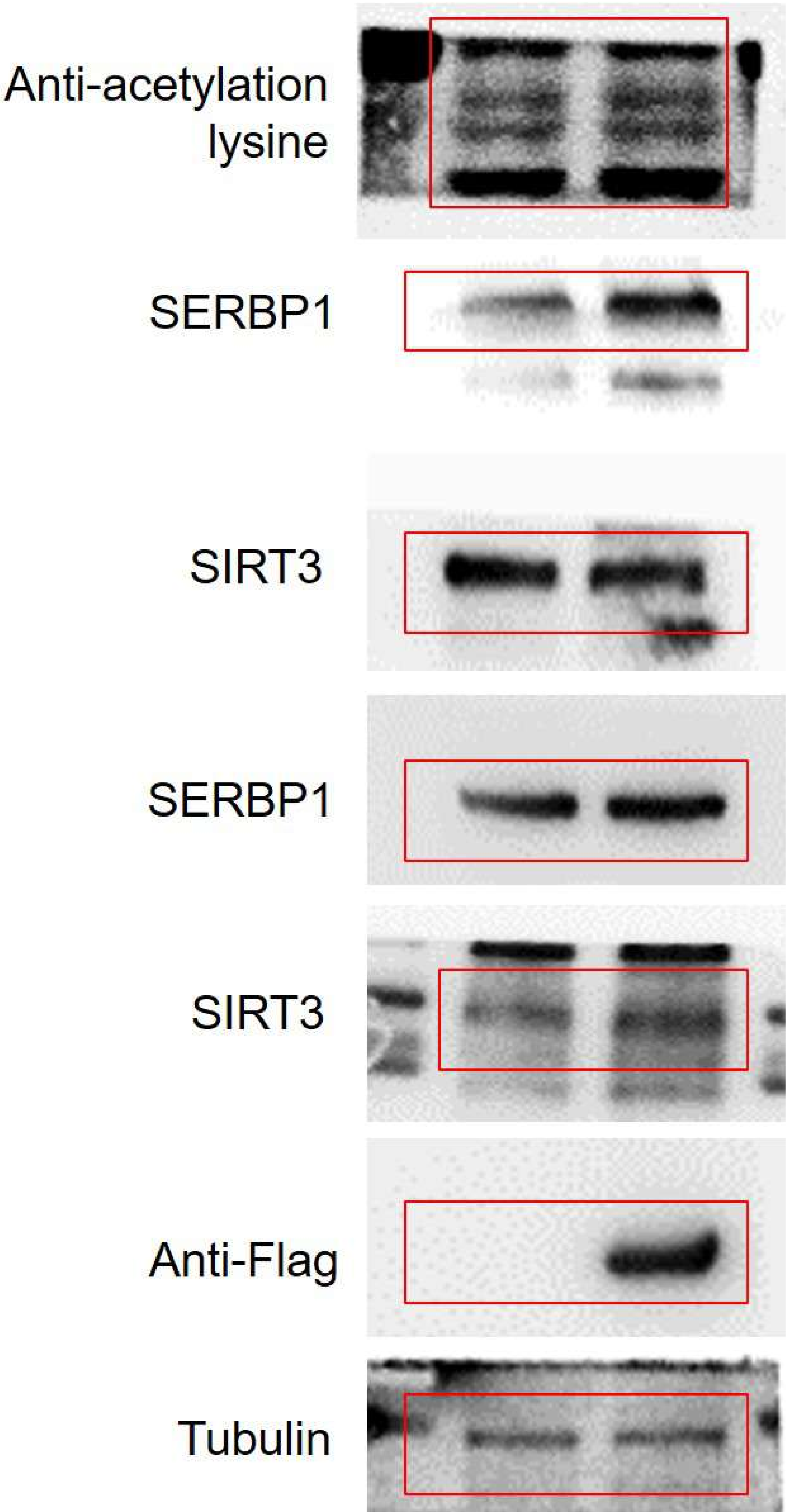
Anti-acetylation lysine
SERBP1
SIRT3
SERBP1
SIRT3
Anti-Flag
Tubulin

Figure 5E

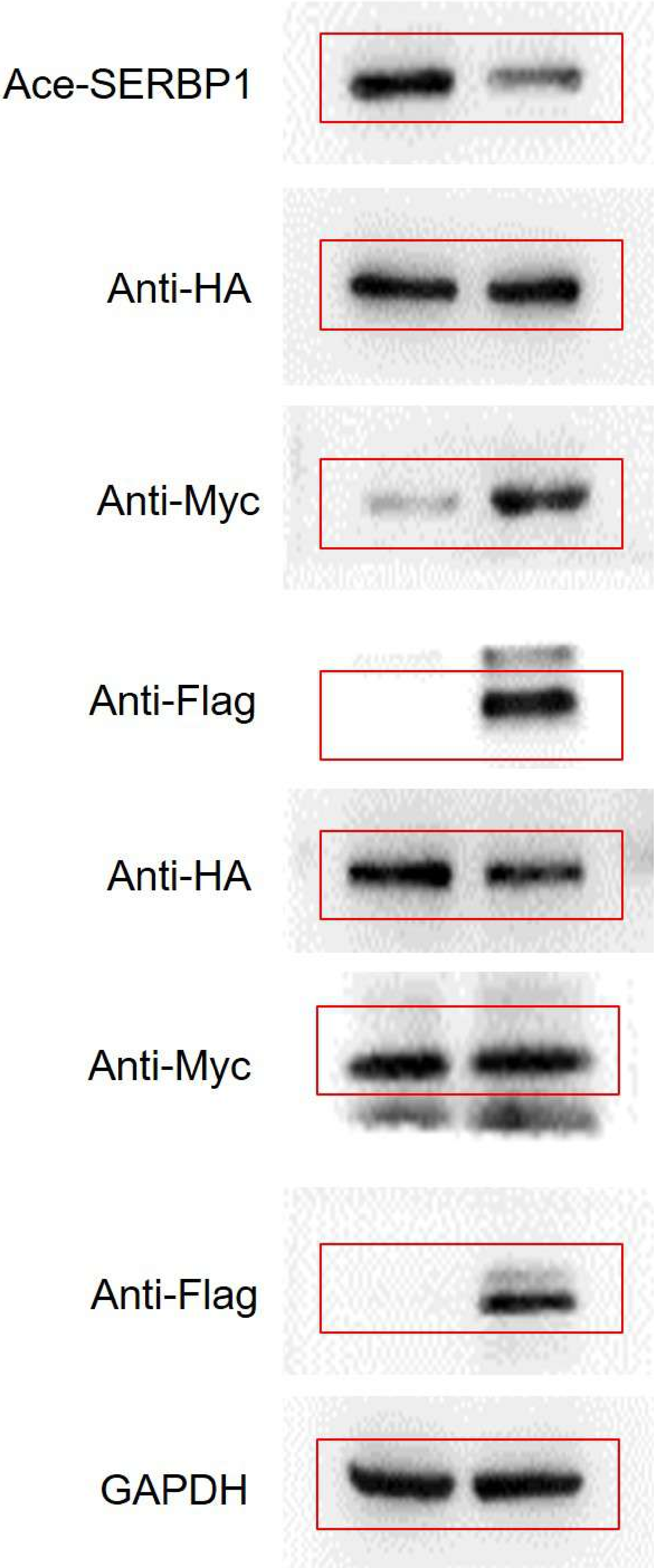
Ace-SERBP1
Anti-HA
Anti-Myc
Anti-Flag
Anti-HA
Anti-Myc
Anti-Flag
GAPDH

Figure 5F

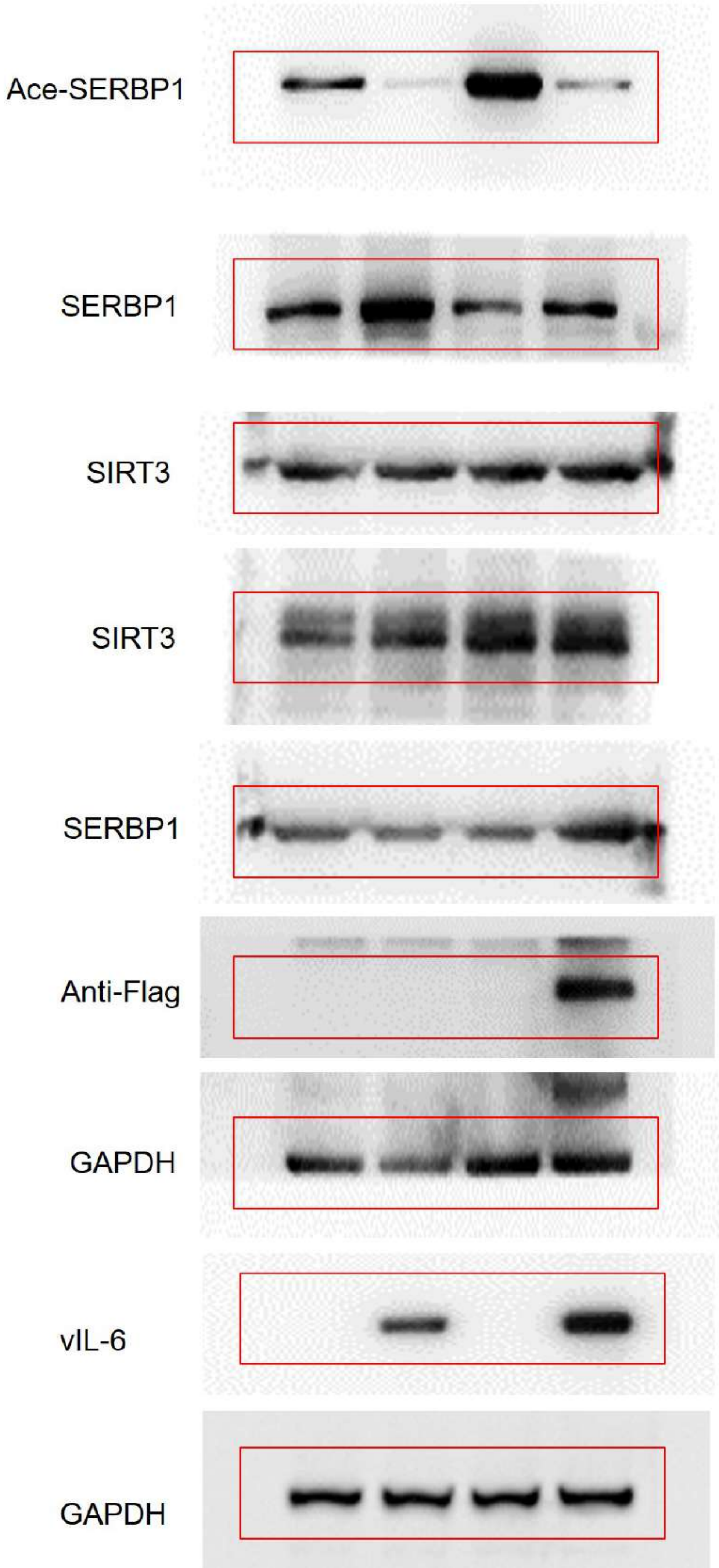
Ace-SERBP1
SERBP1
SIRT3
SIRT3
SERBP1
Anti-Flag
GAPDH
vIL-6
GAPDH

Figure 6F

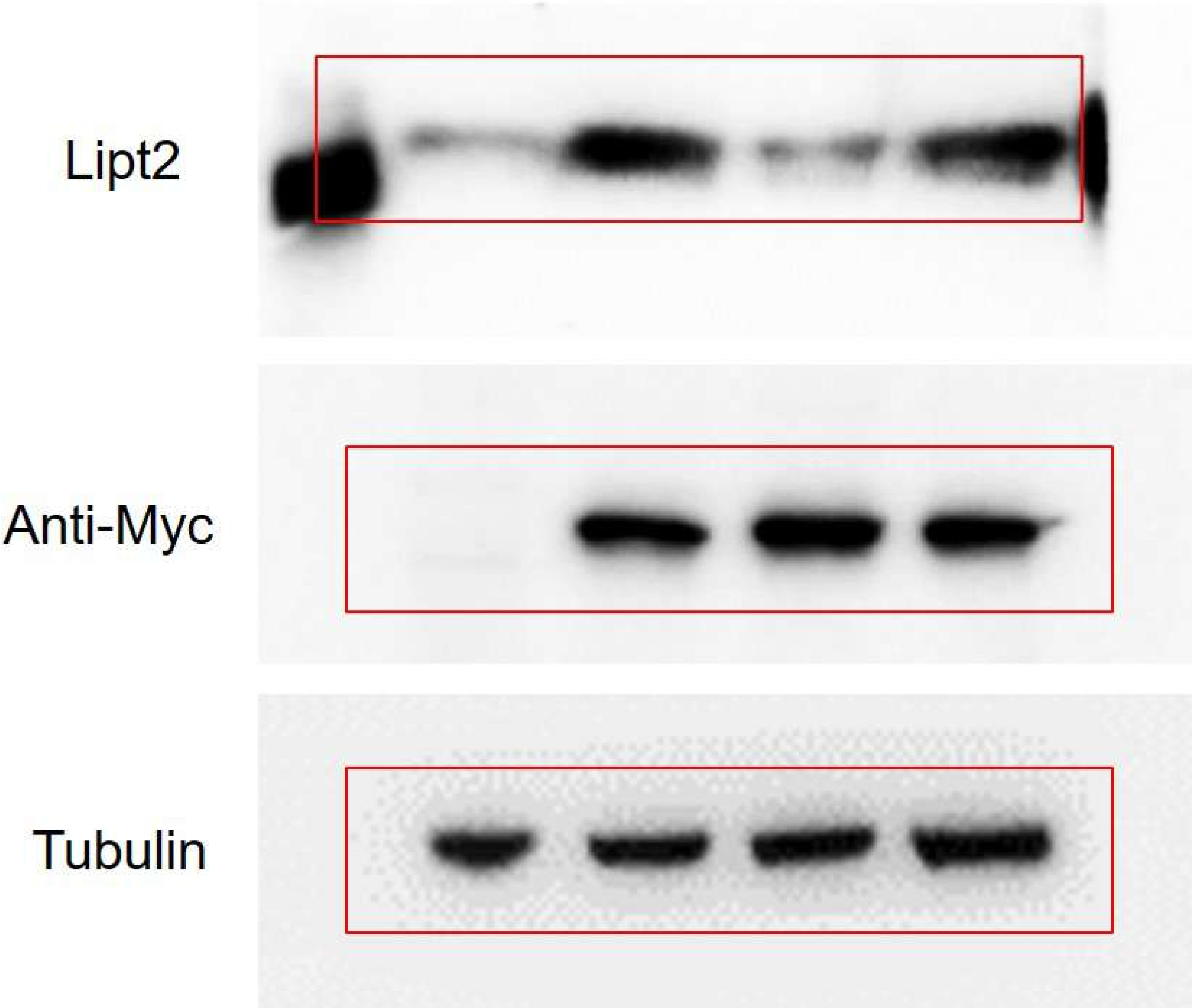

Figure 6J

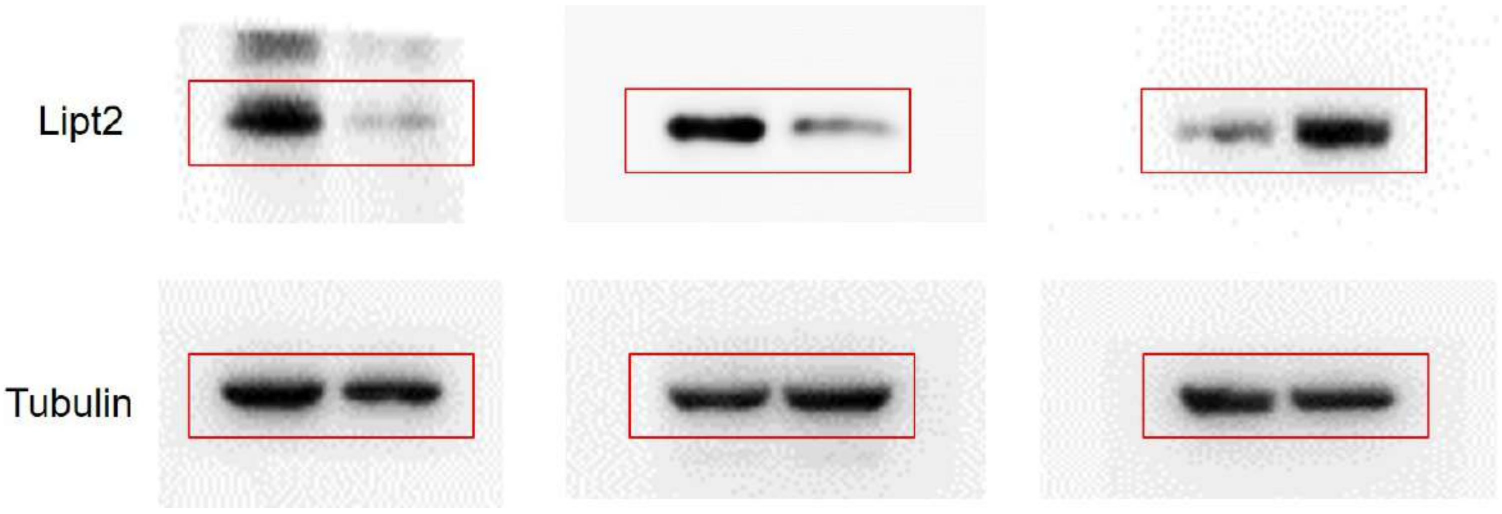

Figure 7E

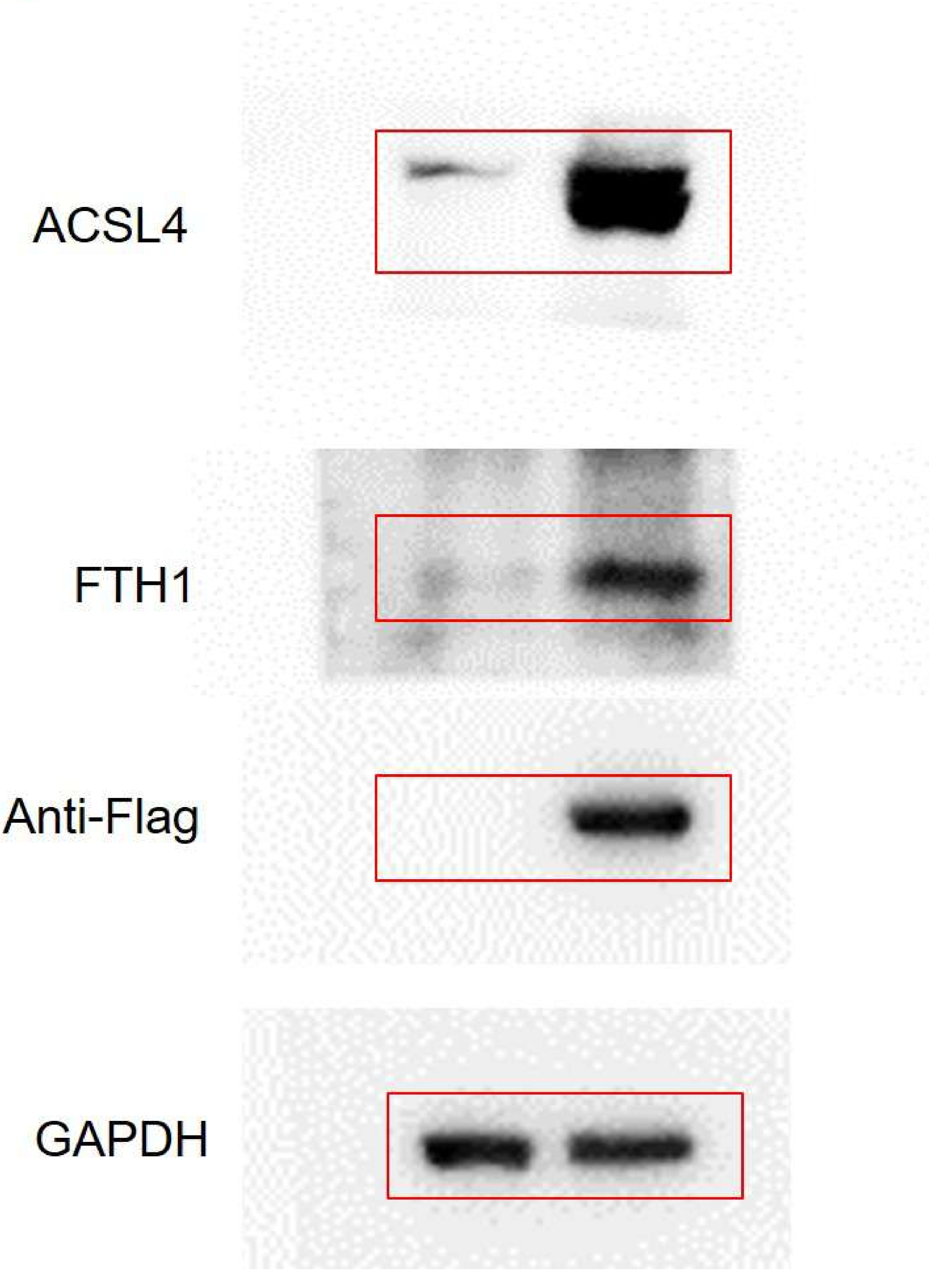

Figure S3A

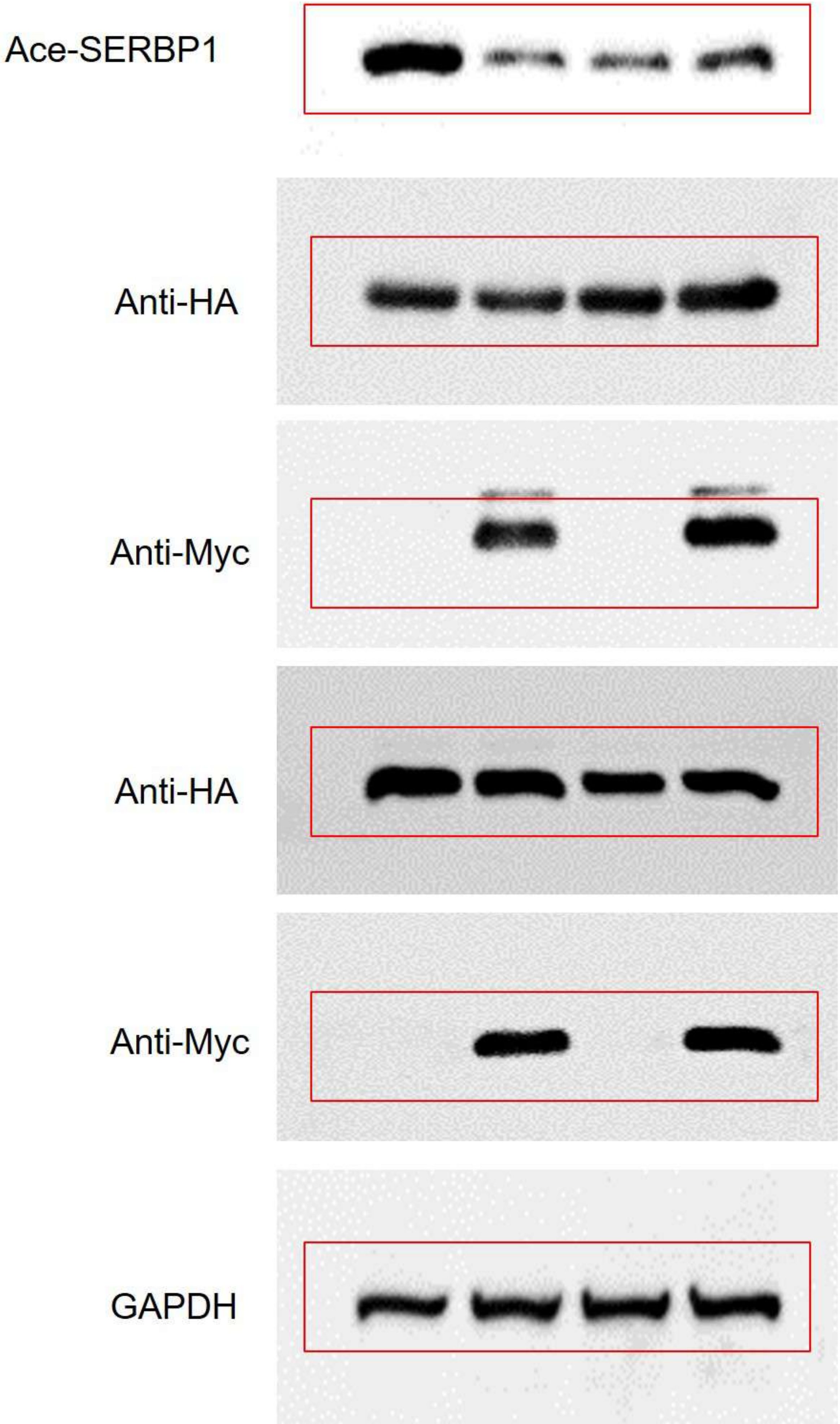

Figure S4A

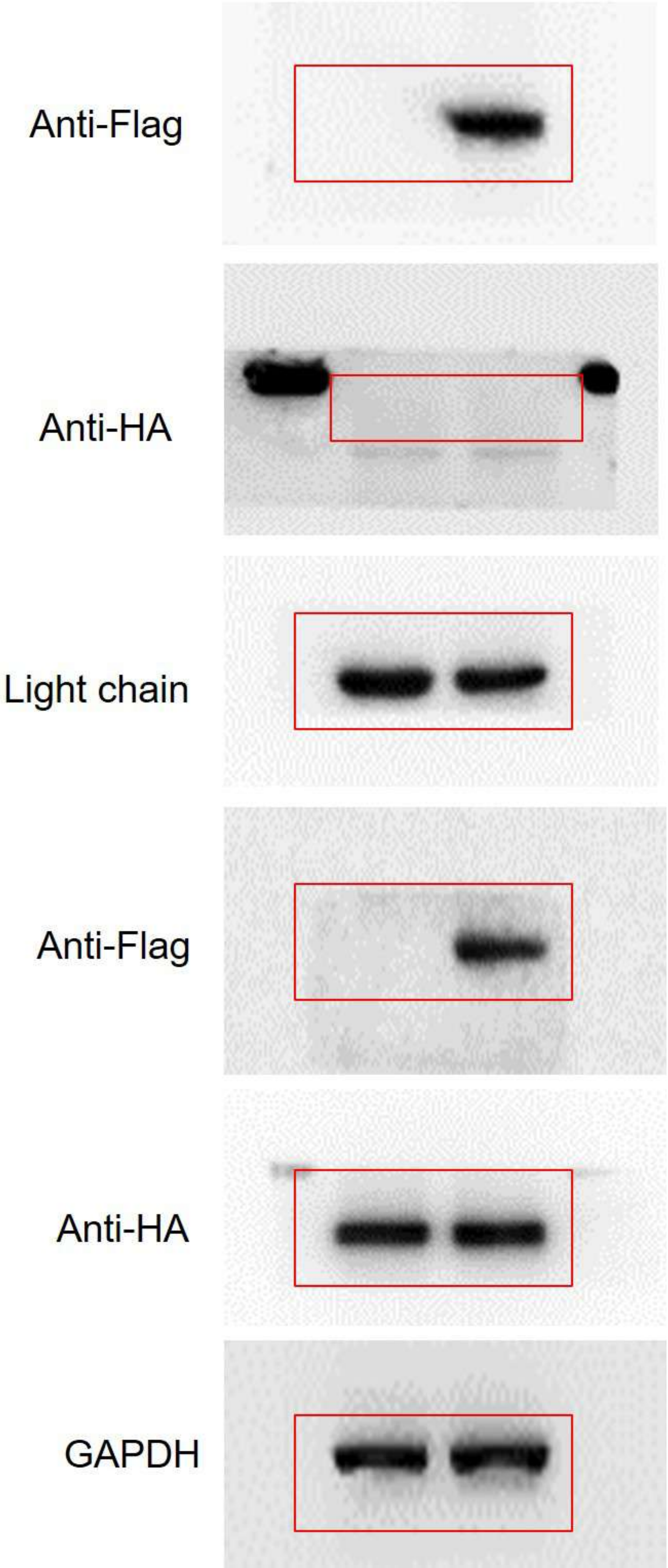

Figure S4B

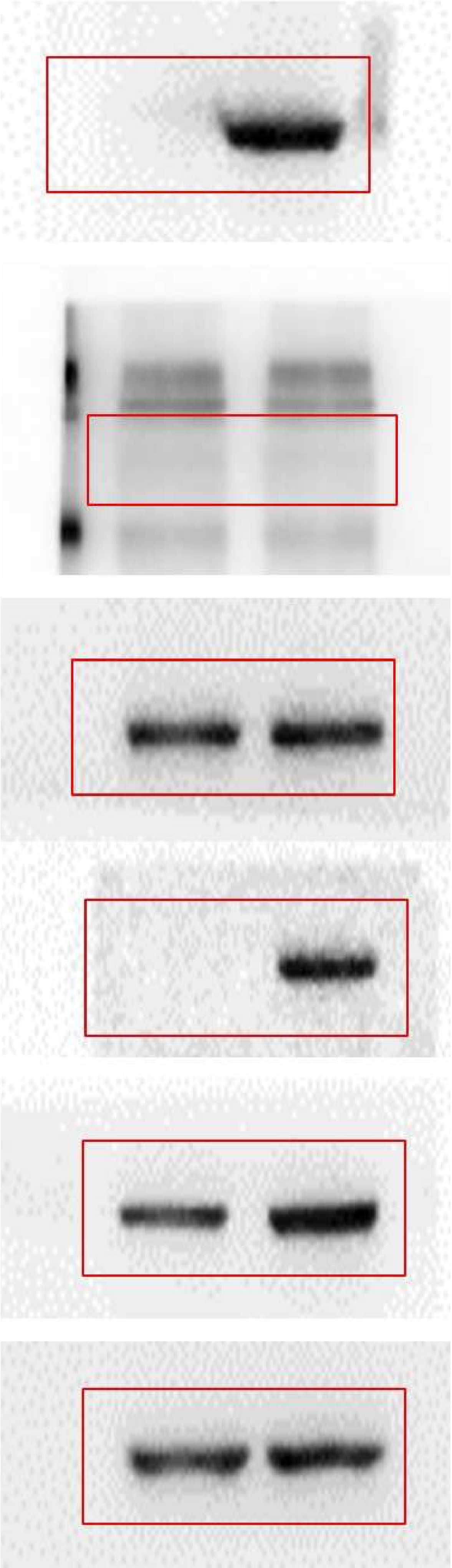

Anti-Myc

Anti-HA

Light chain

Anti-Myc

Anti-HA

GAPDH

Figure S4C

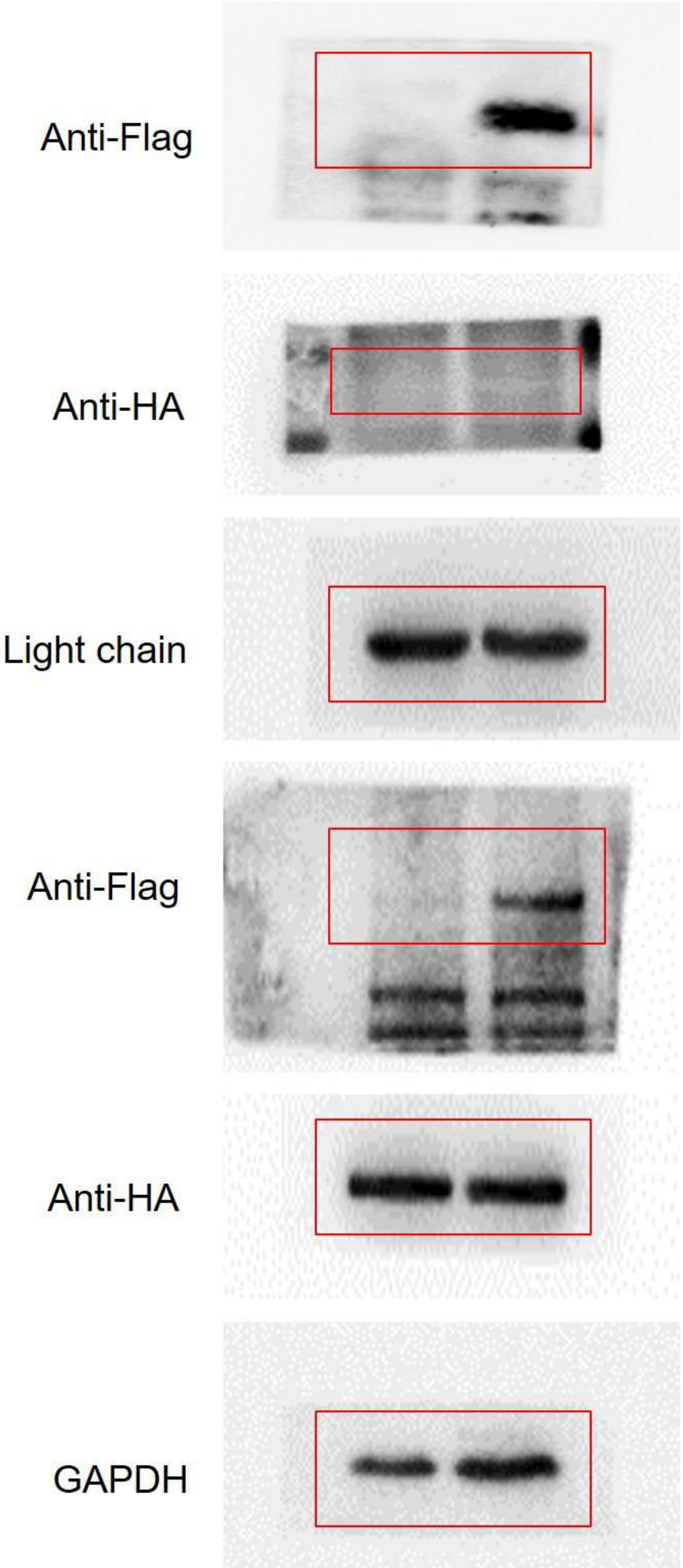

Supplement: S1 Data — (PDF) [file ppat.1012082.s007.pdf]
